# Genomic insights into local adaptation and vulnerability of *Quercus longinux* to climate change

**DOI:** 10.1186/s12870-024-04942-8

**Published:** 2024-04-13

**Authors:** Pei-Wei Sun, Jui-Tse Chang, Min-Xin Luo, Pei-Chun Liao

**Affiliations:** School of Life Science, National Taiwan Normal University, No. 88 Ting-Chow Rd., Sec. 4, Taipei, 116 Taiwan

**Keywords:** Climate change, Genetic offset, Landscape genomics, Local adaptation, Natural selection, Quercus

## Abstract

**Background:**

Climate change is expected to alter the factors that drive changes in adaptive variation. This is especially true for species with long life spans and limited dispersal capabilities. Rapid climate changes may disrupt the migration of beneficial genetic variations, making it challenging for them to keep up with changing environments. Understanding adaptive genetic variations in tree species is crucial for conservation and effective forest management. Our study used landscape genomic analyses and phenotypic traits from a thorough sampling across the entire range of *Quercus longinux*, an oak species native to Taiwan, to investigate the signals of adaptation within this species.

**Results:**

Using ecological data, phenotypic traits, and 1,933 single-nucleotide polymorphisms (SNPs) from 205 individuals, we classified three genetic groups, which were also phenotypically and ecologically divergent. Thirty-five genes related to drought and freeze resistance displayed signatures of natural selection. The adaptive variation was driven by diverse environmental pressures such as low spring precipitation, low annual temperature, and soil grid sizes. Using linear-regression-based methods, we identified isolation by environment (IBE) as the optimal model for adaptive SNPs. Redundancy analysis (RDA) further revealed a substantial joint influence of demography, geology, and environments, suggesting a covariation between environmental gradients and colonization history. Lastly, we utilized adaptive signals to estimate the genetic offset for each individual under diverse climate change scenarios. The required genetic changes and migration distance are larger in severe climates. Our prediction also reveals potential threats to edge populations in northern and southeastern Taiwan due to escalating temperatures and precipitation reallocation.

**Conclusions:**

We demonstrate the intricate influence of ecological heterogeneity on genetic and phenotypic adaptation of an oak species. The adaptation is also driven by some rarely studied environmental factors, including wind speed and soil features. Furthermore, the genetic offset analysis predicted that the edge populations of *Q. longinux* in lower elevations might face higher risks of local extinctions under climate change.

**Supplementary Information:**

The online version contains supplementary material available at 10.1186/s12870-024-04942-8.

## Introduction

Trees profoundly influence global carbon cycles and ecosystem stabilization [1–3]. The effects of anthropogenic climate change on precipitation and temperature patterns have altered the distribution, community composition, and phenology of forest trees worldwide [4–7]. However, the impacts of climate change on focal species vary [8–12]. For example, warming temperatures may have a positive effect on the growth rate of trees from colder environments but a negative effect on trees from tropical regions [10, 13]. Higher temperatures and increased aridity are predicted to increase stress reactions in forest trees under drought conditions, especially tropical and subtropical tree species [14–17].

Studies of the potential response to climate change based on field experiments have emphasized the importance of phenotypic variation for adaptation to local climate and acclimation to drought and cold stresses [18, 19]. However, field experiments are unrealistic for species with long lifespans, such as trees, or endangered species with limited populations. An alternative approach is genotype–environment association (GEA) analysis, which identifies environmental factors that select for genetic characteristics [20, 21]. By predicting the vulnerability of forest trees to climate change and environmental factors that may restrict future distribution, GEA analyses can provide a foundation for conservation projects and forest management [22–24].

Although GEA studies have implied that standing genetic variation may enable some tree species to cope with climate change, the long lifespans and low germination rates of tree species may limit the pace of adaptation to acute and drastic environmental changes [25]. Moreover, the applicability of the results of GEA studies to conservation projects may be confounded by several factors. For example, the geographical distance between source and sink populations and the composition of natural barriers to gene flow may influence pollen dispersal direction and germination rates for introgression between populations [26]. These factors could reduce the spread of beneficial alleles (i.e., genetic rescue effects) [27]. Incorporating landscape analyses into GEA studies could improve the evaluation of genetic vulnerability, assessment of vulnerable populations, and inference of potential dispersal routes under climate change [28, 29].

The tree family Fagaceae is ecologically and economically important and has been widely studied in landscape genetics research [30, 31]. The varied natural habitats of Fagaceae species offer ideal study systems for exploring the effects of environment on genetic diversity, local adaptation, and the response to climate change [32, 33]. Studies have shown that environmental factors, such as precipitation and temperature, significantly affect the adaptive divergence of Fagaceae species [34], but the impacts of other abiotic factors, such as wind, topology, and soil, have largely been ignored [35, 36]. Field experiments have shown that wind and soil factors are prominent drivers of local adaptation of plant morphology and physiology [37, 38], but the potential impacts of these factors on genomic architecture and phenotypic variation across the heterogeneous landscape of Fagaceae species have not been comprehensively established [19, 37, 39, 40]. This is particularly true for Fagaceae species on the subtropical island of Taiwan, where the mountainous terrain and diverse climate have created a range of habitats [41–43].

The rapid development of novel analytic methods in landscape genomics provides unprecedented opportunities to examine new hypotheses and assess vulnerable populations using different statistical assumptions in the context of climate change [2]. If climate change disrupts the allele frequencies that underlie current genetics–environment relationships, vulnerable populations may become less resilient or even extinct locally [44–47]. In this study, we adopted *Quercus longinux* Hayata, an endemic Fagaceae species in Taiwan, as the study species to evaluate the complex effects of environment on local adaptation and the response to climate change. *Q. longinux* inhabits mountainous ranges across Taiwan, from low to middle elevations (500 m to 2,200 m above sea level) [48]. Based on its wide distribution along latitudinal and altitudinal gradients, *Q. longinux* is classified into three varieties (i.e., var. *longinux*, var. *kuoi*, and var. *lativiolaciifolia*), and large morphological variations in fruits and leaves between habitats have been observed [48]. *Q. longinux* var. *longinux* and var. *lativiolaciifolia* grow sympatrically at low to middle elevations in Taiwan. By contrast, *Q. longinux* var. *kuoi* is allopatric with the other varieties and is limited to southeastern Taiwan. Compared with the other two varieties, *Q. longinux* var. *kuoi* has longer, broader leaves that are green on both sides when fresh and have a non-violet abaxial surface when dried [48]. Environmental variations may influence several leaf traits associated with photosynthetic efficiency and acclimation to abiotic stresses [49–51]. Identifying variations in not only genetic data but also leaf morphology that are associated with environment could shed light on the processes that gave rise to the unique *Q. longinux* var. *kuoi* in southern Taiwan.

The ecological function of oaks in subtropical forests is essential, and it will be further important to evaluate the adaptation and their vulnerability when climate change is believed to alter their distribution and deteriorate their survival. The first step to conservation is revealing their population structure and adaptation pattern and identifying potential genetic sources and vulnerable populations. Therefore, this work was guided by three objectives. First, we aimed to infer genetic structure and assess the consistency of relationships between genetic data, ecological niches, and phenotypic traits for *Q. longinux* var. *kuoi*, a special allopatric variety limited to southern Taiwan. Second, we aimed to identify environmental features that affect spatial genetic variation and adaptive genetic divergence. Third, we aimed to use adaptive genetic variants to evaluate the vulnerability of populations to climate change.

## Materials and methods

### Sampling, sequencing, read mapping, and variant calling

All samples analyzed in this study were collected and identified by the authors. We collected 26 populations spanning all known distributional regions of *Q. longinux* in Taiwan (Fig. S1a; Table S1). Three populations in Southern Taiwan (SK, LZ, and GS) were morphologically identified as *Q. longinux* var. *kuoi;* three populations (TP, NS, JSY) were collected near the locations with documented *Q. longinux* var. *lativiolaciifolia* [48]; and the rest populations were identified as *Q. longinux* var. *longinux*. From each selected tree, a branch with more than ten mature leaves was collected for morphological measurements. Fresh leaves were stored in silica gel at 4 °C until DNA extraction. Voucher specimens of this study have been deposited at National Taiwan Normal University Herbarium (TNU) under deposition number TNU057201–TNU057214.

DNA was extracted using the modified CTAB method based on Doyle [52]. DNA quality and quantity were evaluated with a NanoDrop 2000 Spectrophotometer (Thermo Fisher Scientific, Wilmington, DE, USA) and Qubit 2.0 Fluorometer (Life Technologies, Carlsbad, CA, United States), respectively. After quality control, DNA from 205 individuals was used for dd-RAD library construction. The library was double digested by *Sbf1* and *Msp1* and ligated to Illumina sequencing adapters with individual barcodes and library indices. Fragments of 250–500 bp were selected and amplified by polymerase chain reaction (PCR). Finally, the fragments were sequenced using Illumina HiSeq X (Illumina, USA) to generate 150-bp paired-end reads, at the Technology Commons in College of Life Science (National Taiwan University, Taiwan).

We trimmed the raw reads to remove adapters, reads < 50 bp, and reads with quality < 30 using fastp [53]. The clean reads were mapped to the reference genome of *Q. robur* [54] using the mem algorithm of BWA [55]. Aligned reads with a mapping quality score < 20 were discarded. PCR duplicates were marked with the Picard Toolkit (http://broadinstitute.github.io/picard/). We further performed realignment around indels using ABRA2 [56]. Summary statistics, including read number, mapping rate, and coverage, were calculated from SAMtools [57]. Variants were called with BCFtools [58]. We first generated genotype likelihoods at each genomic position with coverage using the BCFtools mpileup command and called the variants with default parameters. After removing indels, we retained biallelic SNPs with missing rates < 0.4, minor allele frequencies > 0.01, genotypes with quality scores > 30, and mean minimum depths > 3 as filtering thresholds in VCFtools [59].

### Genetic diversity, genetic structure, and demographic analysis

To evaluate and visualize the genetic clusters of *Q. longinux*, we first performed principal component analysis (PCA) using a genetic matrix in which missing data were replaced with mean values of each site in the R package adegenet [60]. Next, pairwise *F*_ST_, population-specific *F*_ST_, and permutational multivariate analysis of variance (PERMANOVA) were performed with the R packages hierfstat [61] and vegan [62] to evaluate differentiation among defined genetic clusters. A neighbor-joining (NJ) tree was also constructed based on the Nei distances from the R package ape [63]. We then employed StrAuto [64] analysis to assess the pattern of admixture among populations using 100,000 steps of MCMC chains with 25,000 steps burn-in and 10 iterations from *K* = 1 to 8. The results were uploaded to the CLUMPAK [65] server to generate consensus plots and evaluate the best *K* according to the Δ*K* value.

A stairway plot was used to infer historical changes in the effective population size (Ne) of *Q. longinux* [66]. We first generated the site frequency spectra (SFSs) from easySFS (GitHub repo: https://github.com/isaacovercast/easySFS) using all SNPs and selected the projection values with the highest segregating sites for each group. We then ran bootstrapping 1,000 times to estimate median sizes and 95% CIs, assuming a mutation rate of 1.01 × 10^− 8^ per bp per generation and a generation time of 50 years for oak species [67].

To provide an in-depth investigation of the evolution history between *Q. longinux* var. *kuoi* and the rest of the populations, we simulated and compared four scenarios (Fig. S1b) with different patterns of migration using fastsimcoal2 [68]. All models were simulated and optimized using the composite likelihoods calculated with SFSs. Because the computation of fastsimcoal2 is demanding, observed SFSs were projected to five individuals per simulated population. A mutation rate of 1.01 × 10^− 8^ per bp per year was assumed for estimating all parameters with a generation time of 50 years [67]. To select the best model, we first used the Akaike information criterion (AIC), which represents the difference between the observed and expected SFSs, to rank the optimized models. Second, the parametric-bootstrap approach was used to calculate 100 likelihoods and the 95% CI for the parameters estimated by the best models and to select the best-fitting models according to the lowest bootstrapping AIC.

### Environmental variables and GIS processing

Three categories of environmental variables were collected: 105 climate variables, eight soil variables, and four topological variables (Table S2). The climate variables included BIO01-19, seven monthly precipitation factors, temperature, solar radiation, and wind speed downloaded from WorldClim2 [69] and two aridity-associated layers [70]. Eight physicochemical soil characteristics downloaded from SoilGrids 2.0 were used as the soil variables [71]. For topography, the altitude layer was downloaded from GOV.DATA.TW (https://data.gov.tw/), and three altitude-derived layers (i.e., roughness, aspect, and slope) were computed with the R package raster [72]. All variables were resampled to a resolution of 1 km^2^ for the downstream analyses. To address the issue of multicollinearity, variables with VIF > 10 and Pearson correlation coefficient |r| >0.8 were discarded, leaving 20 environmental variables among the three categories (Table S3; Supplementary data) for use in the subsequent analyses. We compared differences in the environmental variables between groups using analysis of variance (ANOVA) and Tukey’s honestly significant difference test applied in the R package stats [73]. The boundaries of Taiwan and nearby islands were downloaded from GADM database (http://www.gadm.org/). All maps in this study were depicted by the authors using R 4.2.3 [73].

### Assessment of ecological and morphogenetic divergence

#### Niche differentiation assessment

To harness the power of GEA analysis to evaluate a species’ ability to cope with future climate change, it is crucial to include non-genetic factors that may be associated with fitness to local environments, such as niche characteristics and phenotypic traits. To investigate and quantify the niche overlap and diversification between the three (eastern, western, and southern) genetic groups revealed in our results, we first assessed the overlap of the realized niche using the first two axes from the environmental PCA performed with the 20 retained environmental variables in the R package ecospat [74]. Five hundred pseudo-absence points were added to the analyses as background. In addition, we calculated two niche overlap indices: Schoener’s *D* and the standardized Hellinger-transformed Warren’s *I* based on occurrence density grids. Next, we conducted the equivalency test with 100 permutations and the background test with 100 runs to evaluate the significance of niche differentiation at *p* < 0.05. The equivalency test and background test were implemented with the R package ENMtools [75].

#### Phenotypic trait analysis

To quantify the contributions of genetic and environmental factors to phenotypic variations, we measured 12 morphological leaf traits that are commonly used in morphological studies of Fagaceae and other plants (Table S3; Fig. S2; Supplementary data) [76–78]. A total of 826 dried leaves of 191 individuals from 26 populations (1–5 leaves per individual) were recorded with a digital camera and measured using ImageJ [79]. Because the measured traits may be highly correlated with leaf size, an additional six computational traits that are not typically correlated with leaf size were added [78]. We first performed PCA and PERMANOVA with the six computational traits (i.e., shape-related traits and specific leaf area (SLA)) and abaxial surface color to quantify morphological differences among groups using the R package vegan [62]. Biplots of the attributes and contributions were visualized with the R package factoextra [80]. Differences in traits between groups were compared using ANOVA and Tukey’s post hoc test. Next, we performed partial redundancy analysis (RDA) to evaluate the contributions of geography, demography, and environment to phenotypes using all scaled phenotypic traits as the response.

To further investigate the associations between morphological variables and environmental predictors, we constructed univariate generalized linear models (GLMs) with one of the leaf traits as the response (Table S4) and one of the environmental variables as the predictor in the R package MASS [81].

### Detection of local adaptation and underlying gene functions

#### Identification of environment-associated genetic variants

We integrated two *F*_ST_-based outlier detection methods and one genotype–environment association (GEA) approach to detect environment-associated SNPs. First, we implemented BayeScan [82] and pcadapt [83] to identify SNPs with a significant departure of allele frequency from neutrality while controlling for background genetic structure. BayeScan was run with posterior odds (PO) = 100, and SNPs with *q* < 0.01 were retained as putative outliers. Pcadapt was performed with the number of *K* principal components (PCs) selected by the decreasing order of the percentage of variation explained by each PC. We further implemented Benjamini–Hochberg correction [84] on the results and retained the SNPs with a false-discovery rate (FDR) < 10% as candidate outliers.

Second, we utilized a univariate approach, the latent factor mixed models (LFMM) algorithm [85, 86], to discover SNPs that were significantly correlated with environmental variables while accounting for population genetic structure. Imputation of missing data and determination of latent factors (*K*) were performed by the snmf function in the R package LEA [85]. LFMM was performed in the R package LEA [85] using the retained environmental variables with ten runs, and *Z*-scores from each run were combined. To further adjust the *p*-values, the Benjamini-Hochberg procedure [84] was used, and FDR < 10% was used to identify putative adaptive outliers. SNPs that overlapped between significant environment-associated outliers and the BayeScan or pcadapt outliers were regarded as putative adaptive SNPs for use in downstream analyses.

To investigate and compare the roles of geography, demography, and environment in shaping the genetic variation of adaptive and all SNPs, we decomposed the relative contributions of each group of predictors using three methods. First, Mantel tests were used to test for associations between *F*_ST_/(1-*F*_ST_) and geographic distance (isolation by distance, IBD) and environmental distance (isolation by environment, IBE) using the R package vegan [62]. Environmental distances were represented by the Euclidean distances of scaled climatic, soil, and topographical variables. Values of pairwise *F*_ST_ between populations were calculated with the R package hierfstat [61]. Differences in *F*_ST_ between adaptive and all SNPs were compared by ANOVA in the R package stats [73].

Second, we performed partial redundancy analysis (RDA) using three predictor datasets: (1) proxies of geographic structure obtained by converting the geographic coordinates to uncorrelated axes using principal coordinates of neighborhood matrices (PCNM) in the R package vegan [62] and retaining half of the positive axes; (2) proxies of demographic history obtained by performing PCA using the genetic matrix of all SNPs and retaining the PC scores from PC1 to PC2; and (3) the 20 retained environmental variables. The genetic metrics calculated from the adaptive or all SNPs were used as the response variables. To further quantify the variation explained by each group of environmental variables, we performed another partial RDA using the 20 environmental variables, which were classified into (1) climatic, (2) soil, and (3) topographical variables, as predictors of the two genetic metrics as the response. The significance of the full models and pure fractions was assessed using 999 permutations with the function anova.cca() of the R package vegan [62].

Third, we used GradientForest (GF), a non-linear and machine-learning approach derived from the random forest algorithm [87]. GF was performed with the genetic metrics of the adaptive or all SNPs. The number of regression trees was set to 500, the maximum number of splits was set using the formula log_2_ (0.368 × number of individuals/2), and the correlation threshold *r* was set to 0.5, as suggested in a previous study [87]. The importance of each group of predictors was evaluated by the percentage of weighted *R*^2^.

#### Gene annotation and enrichment analysis

To further investigate the potential gene functions of putative environment-associated SNPs, we retrieved the closest genes with a physical distance < 10 Kbps as potential underlying genes using BEDOPS [88]. The closest genes were further annotated to the *Arabidopsis thaliana* genome using KOBAS-I [89] with the criterion of *e*-value < 10^− 5^. After retrieving the homologous genes, gene enrichment analysis was performed with the GO and KEGG databases to detect enriched pathways using KOBAS-I. Significance was assessed with Fisher’s exact test [90], and FDR was corrected with the Benjamini and Hochberg procedure [84]. FDR < 5% indicated significantly enriched GO terms or pathways.

### Investigating the influences of current landscape variables on genetic differentiation

To compare landscape characteristics that shaped the differentiation of adaptive or all SNPs, we constructed IBD, IBE, and isolation by resistance (IBR) models using *F*_ST_/(1-*F*_ST_) calculated from adaptive or all SNPs as a response. Six predictor matrices were generated, including a geographical Euclidean distance metric (IBD), three environmental Euclidean distances calculated from the climate, soil, and topology factors (IBE_climate_, IBE_soil_, and IBE_topology_), and two circuit-theory-based resistance layers calculated with CIRCUITSCAPE [91] using distribution maps of ecological niche modeling from the factors of climate and topography (e.g., IBR_climate_, IBR_topography_).

We used two complementary approaches to compare the models. First, reciprocal causal modeling (RCM) [92] was performed to evaluate the relative importance of each model based on the relative values of partial Mantel tests estimated from each focal and alternative model. All partial Mantel tests were computed using the R package vegan [62] with 999 permutations. Second, we implemented the maximum likelihood population mixed-effects model (MLPE) [93] to compare each predictor based on the AIC values. Models with ΔAIC > 2 were considered to have different model fits. MLPE was implemented with mlpe_rga in the R package ResistanceGA [94]. In addition, we performed an estimated effective migration surfaces (EEMS) analysis [95] to visualize geographic regions deviating from the assumption of IBD. Missing data were imputed and converted to genetic distance using the function bed2diffs. Next, EEMS was executed with RunEEMS_SNPS with a Deme number of 500. A total of 500,000 Markov chain Monte Carlo (MCMC) iterations were run after a burn-in of 150,000 and a thinning interval of 9,999.

### Investigating the impacts of future climate change on genetic adaptedness

#### Ecological niche modeling

To investigate the current potential distribution range of *Q. longinux*, we performed ecological niche modeling (ENM) using an ensemble approach based on the true skill statistic (TSS)-weighted combination of six methods in the R package sdm: maxent, glm, svm, gam, mda, and mlp [96]. Five hundred pseudo-absence data were generated and added to the analyses. A total of 60 runs with 10-fold cross-validation analyses were performed. We then used the area under the receiver-operator curve (AUC) to evaluate model performance. Finally, the habitat prediction was transformed into a binary map that classified suitable and unsuitable regions using the threshold of maximum test sensitivity plus specificity.

#### Genetic offset assessment

Because the accessible prediction layers are limited, we only applied five temperature and precipitation variables (i.e., BIO1, BIO3, BIO12, PREC4, and PREC10) selected from the obtained factors to assess the influence of climate change on *Q. longinux*. We downloaded and averaged the predictions from three future models (CCSM4, MIROC-ESM, and MIROC5) in 2070 to account for model variability. We also considered two contrasting representative concentration pathways (RCPs): a low-emission model (RCP2.6) and a high-emission model (RCP8.5) from CMIP5 for 2070. Three complementary approaches were used to evaluate genetic offset. We first calculated the risk of non-adaptedness (RONA), which represents the theoretical changes needed in allele frequency to maintain the linear environment-genotype relationships with correction on weighted *R*^2^, implemented in pyRONA [97]. We retained and discussed the top three representative climatic variables with the highest number of significant outliers.

Second, as a complementary method to RONA, we used a random forest algorithm to model the non-linear relationships between adaptive SNPs and the same climatic variables as RONA in the R package GradientForest [87]. PCA was performed on the GF model to visualize the prediction of genetic variation in spatial regions, and the first three principal components (PCs) were assigned to a red–green–blue palette. The genetic offset in the GF model was calculated as the Euclidian distance between the current and future genetic composition at each grid. The binary map generated by ENM was used as a mask to limit prediction within suitable habitats.

Third, following Gougherty, Keller [98], we integrated migration to predict maladaptation to future climate using a generalized dissimilarity modeling (GDM) algorithm [2] with local, forward, and reverse genetic offsets. Local offset represents the predicted *in situ* change in allele frequencies with no migration. Forward offset was calculated by selecting the minimum offset between each grid in the current range and all grids in the future climate, and reverse offset was calculated by identifying the minimum offset between each grid within the current range in the future and all grids in the current climate. For forward offset, the distance required to migrate to the place with minimum forward genetic offset and the direction (bearing) of migration were also recorded. No dispersal limitation was assumed for forward and reverse offsets to any locations in suitable habitats. Finally, to simultaneously visualize local, forward, and reverse offsets, we mapped the three metrics as red, green, and blue in an RGB image.

## Results

### Population structure and demographic history

**Fig. 1:**
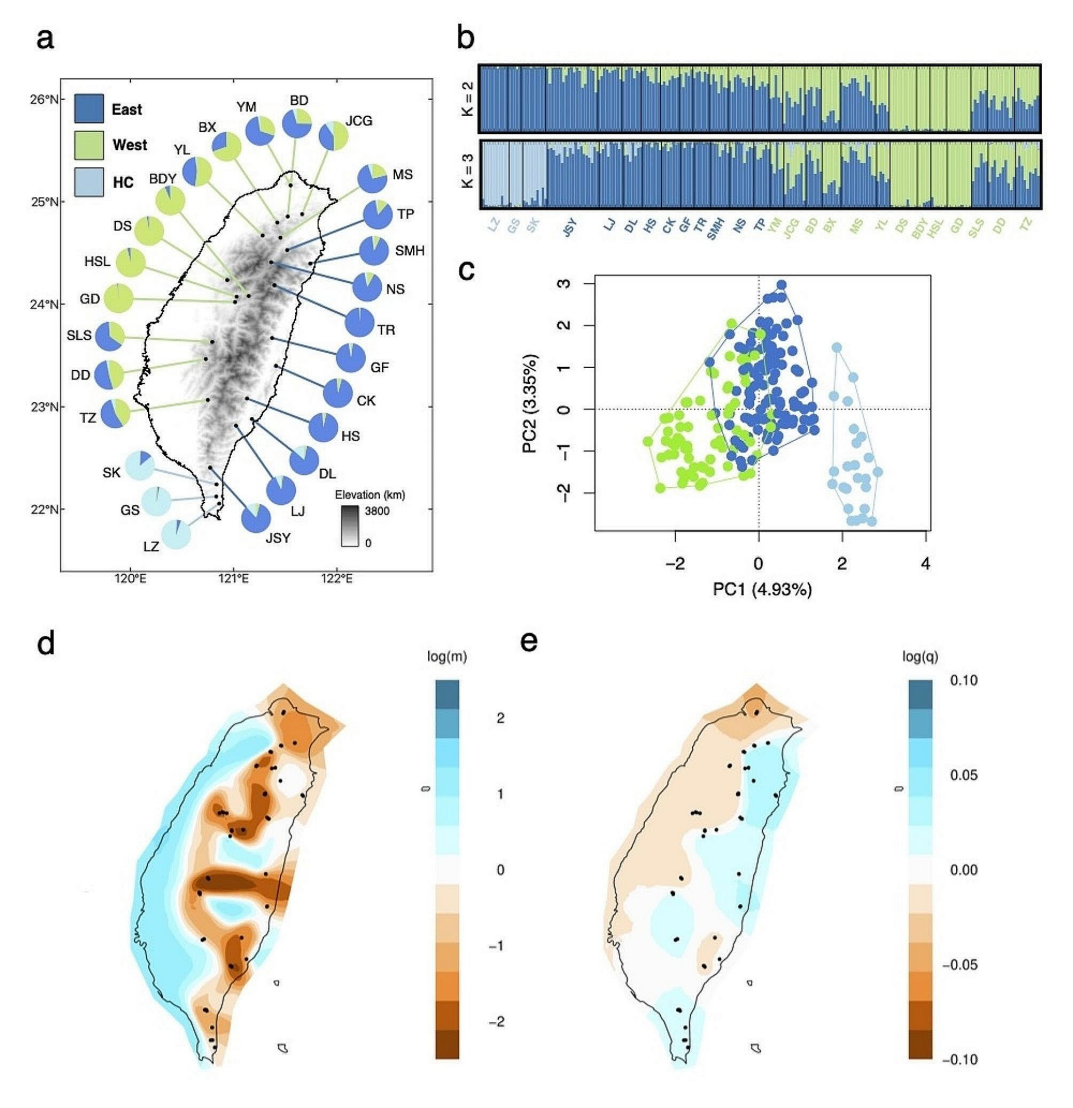
Spatial genetic structure and diversity of *Q. longinux* in Taiwan. (a) Sampling populations annotated with ancestral components inferred by StrAuto according to *K* = 3. (b) Results of StrAuto from *K* = 2 to 3. The height of each colored segment represents the possible ancestry of each individual derived from inferred ancestors. (c) Results of PCA with different colors reflecting different groups. (d) Results of the EEMS analysis of regions significantly deviating from isolation by distance (IBD). The blue, white, and orange colors illustrate regions with high dispersal rates (dispersal corridor), IBD, and low dispersal rates (dispersal barrier), respectively. (e) Results of the effective diversity (i.e., the modeled dissimilarity between pairs of individuals from the same location) estimated by the EEMS analysis. Blue and orange represent regions of high and low genetic diversity, respectively. The black dots indicate the sampling locations

On average, 70.9% of reads per sample were successfully mapped to the reference genome of *Q. robur*. We recovered 624,451 SNPs and retained 1,933 high-quality SNPs with an average individual missing rate of 11%. StrAuto revealed three genetic clusters (Fig. 1a): the eastern and western clusters, which are mainly separated by the central mountain range (CMR), and a cluster in the Henchung Peninsula that was limited to southern Taiwan, denoted as HC. From the result of StrAuto, individuals in each cluster were genetically admixed with other clusters (Fig. 1b). PCA and the neighbor-joining (NJ) tree also suggested that the boundaries of the three clusters were not clearly defined (Fig. 1c; Fig. S3a-b). *F*_ST_ was higher between genetic clusters than within groups, and divergence was highest between HC and the eastern cluster (Fig. S4c). Similar results were obtained by PERMANOVA (Table S5). The results of the estimated effective migration surface (EEMS) analysis also suggested the connectivity and geographic distribution of genetic diversity were mainly separated by CMR (Fig. 1d-e). According to the stairway plot (Fig. S4d), *Ne* has differed between HC and the other two clusters since 1 million years ago (Fig. S4d). The apparent population decline of HC 1 million years ago was preceded by a period of larger population size. By contrast, the eastern and western clusters underwent a relatively recent population decline in the last 100 Ky. The fastsimcoal2 analysis suggested no apparent gene flow between group HC and the eastern and western clusters since their divergence of 19.6 Kya (Table S6). Recent bottleneck events were also revealed at 11.5 Kya from the best models for all simulated populations.

### Influence of landscape factors on population genetic divergence

**Fig. 2:**
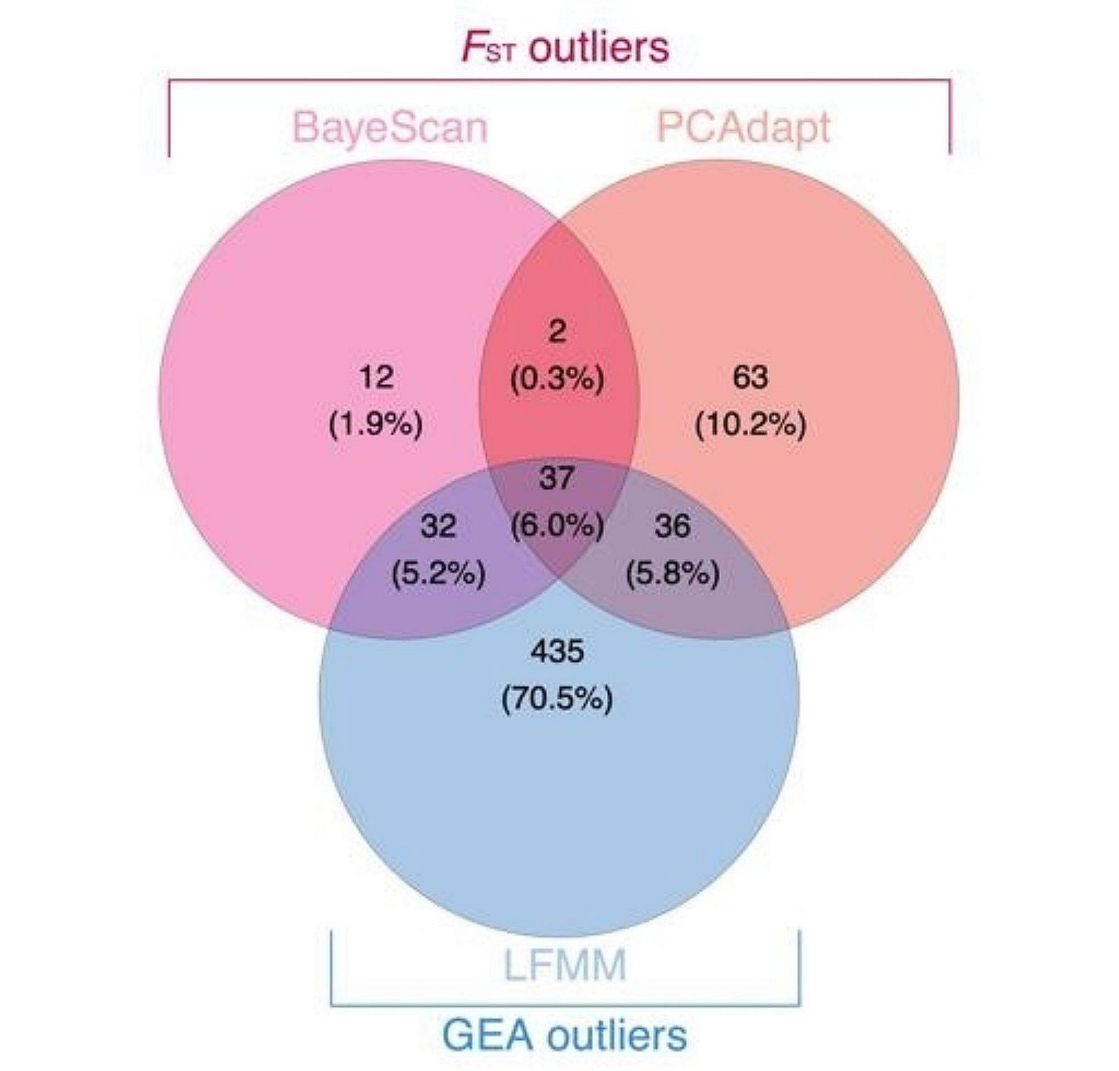
Results of the genetic scans. The Venn diagram shows the overlap of the loci identified by each method

**Fig. 3:**
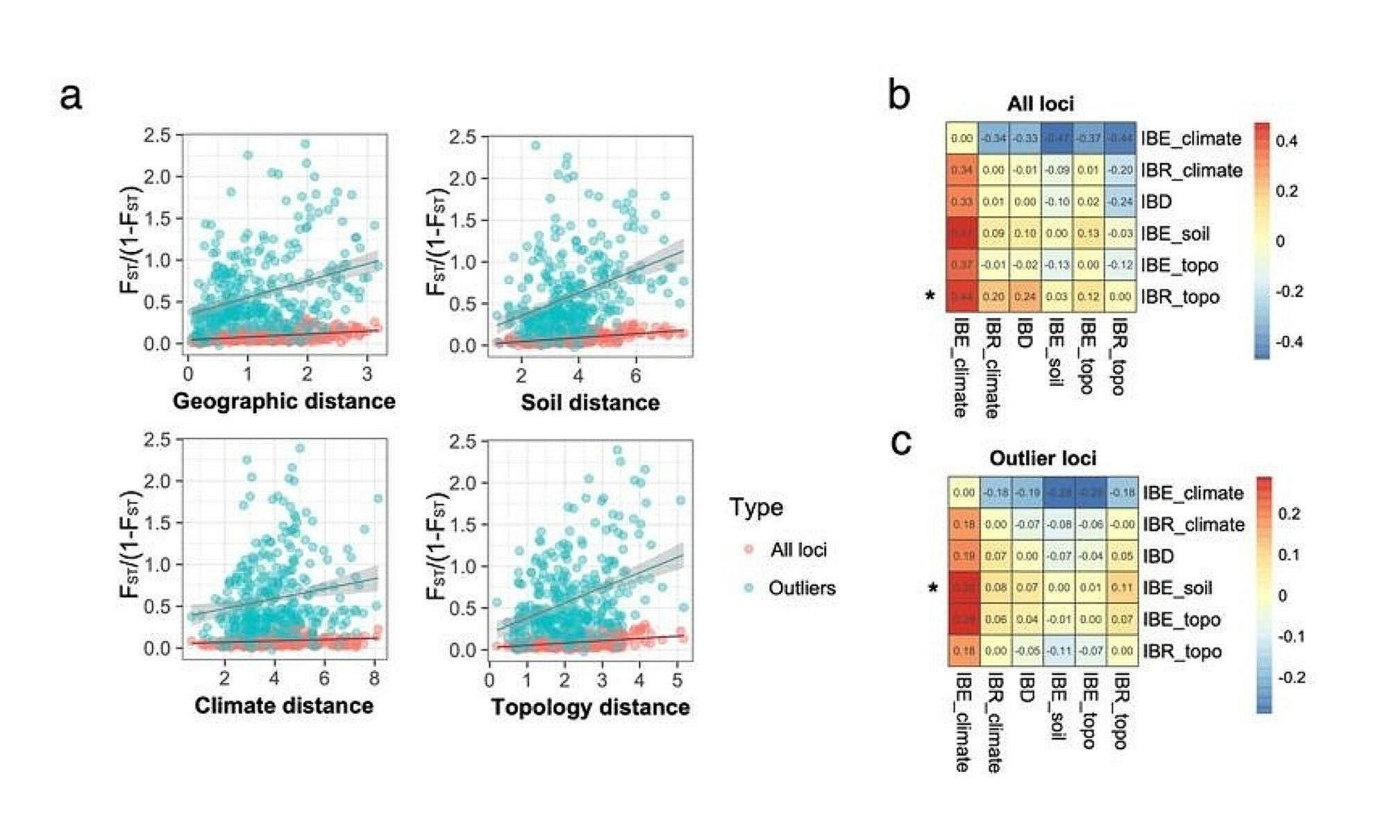
Landscape genetics of *Q. longinux* and factors influencing genetic divergence. (a) Isolation by distance (IBD) analysis and three isolation by environment (IBE) analyses based on adaptive sites (green dots and upper lines) or all SNP sites (red dots and lower lines). (b) Results of reciprocal causal modeling using all SNP sites or (c) adaptive loci. The y-axis represents focal models, and the x-axis represents alternative models. The colors of the heatmap indicate differences in Mantel’s R between the focal and alternative models. Fully supported focal models are marked with asterisks

We identified 182 *F*_ST_ outlier SNPs (pcadapt + BayeScan) and 540 SNPs that were significantly associated with one or more environmental variables by LFMM (Fig. 2). A total of 105 putative adaptive SNPs (identified by the overlap between LFMM and *F*_ST_ outliers) were found in 35 genes (Table S7) that were widely distributed across the genome and did not cluster in specific regions. *F*_ST_ estimate from adaptive SNPs was higher and more significant than all SNPs (mean *F*_ST_ all loci = 0.09; mean *F*_ST_ outlier = 0.59; *p* < 0.05), indicating that selection may drive the spatial genetic differentiation between populations. Compared to all loci, the adaptive loci exhibited stronger IBD and IBE patterns (Fig. 3a). Using reciprocal causal modeling (RCM) and a maximum likelihood population mixed-effects model (MLPE), IBE was consistently identified as superior to other competitive models using adaptive SNPs when controlling population structure, indicating that genetic differentiation of the adaptive SNPs was mainly influenced by environmental variation (Fig. 3b-c; Table S8). Furthermore, the divergence of the genetic structure of HC from those of the other clusters was greater when divergence was assessed by adaptive SNPs than by neutral SNPs (Fig. S4a-b). By contrast, IBR based on topographical resistance was selected as the best model by MLPE and RCM using all sites, suggesting that the overall genetic differentiation of the species was mainly influenced by topographical dispersal barriers (Fig. 1d-e; Table S8). The conductance layers generated by the CIRCUITSCAPE algorithm also supported topographical resistance across regions, with greater barriers to dispersal at higher elevations (Fig. S4e-f).

The results of partial RDA indicated that pure environment was the most significant variable affecting the genetic variation of adaptive SNPs among three pure-effect portions; whereas pure demography was the most important pure-effect portion of all SNPs when controlling other factors (Fig. S5; Table S9). Forward selection also identified population structure as the most significant variable affecting the genetic variation of adaptive SNPs (Table S10). Environmental variables contributed to 80% of the explained variation in adaptive loci but 60% in all loci (Fig. S5). For adaptive loci, the three groups of variables explained a large proportion of the joint effects (45% of explained variation) (Fig. S5; Table S9). Another partial RDA that decomposed the contributions of climate, soil, and topography to the genetic variation in either the adaptive or all SNPs revealed that pure climate and soil variables explained significantly more of the genetic variation in adaptive SNPs than topography variables (Fig. S5). In agreement with the results of partial RDA, GF analysis demonstrated that demography was the top variable with the highest weighted *R*^2^ (Table S11; Fig. S6), although geography explained a higher proportion of variation after summing the total weighted *R*^2^ (demography: 32%; geography: 47%).

### Functional annotation of adapted loci

Several *Q. longinux* loci involved in climate and soil adaptation were associated with environmental variables (Table S7). Two functional pathways were significantly enriched in adaptive loci: oxidative phosphorylation and photosynthesis.

**Fig. 4:**
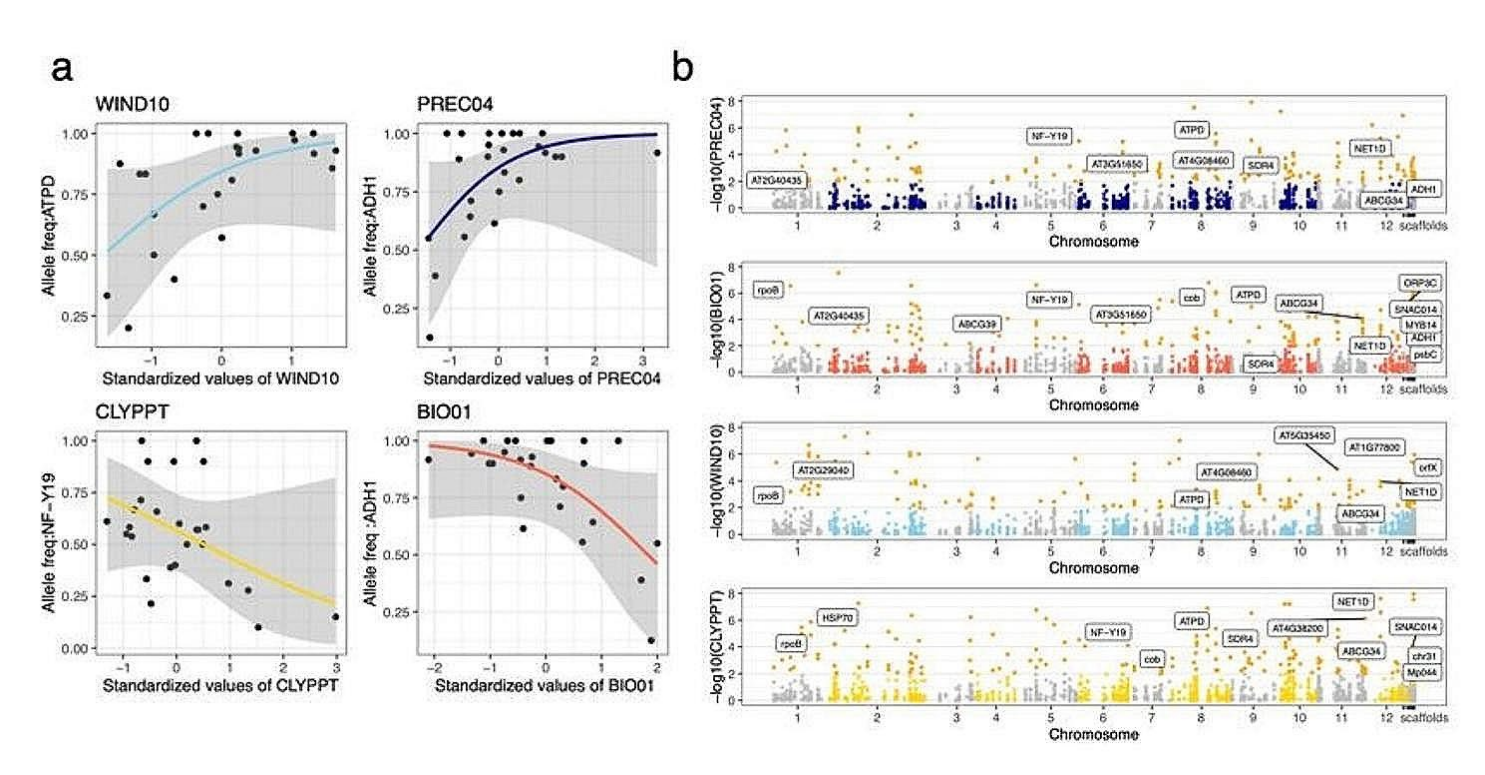
Results of environment-dependent outlier detection. (a) Associations between allele frequencies and environmental variables. The black dots represent the average allele frequencies of all adaptive loci correlated with WIND10, PREC04, CLYPPT, and BIO01 for each population. (b) Manhattan plots of SNP sites associated with WIND10, PREC04, CLYPPT, and BIO01. The orange dots represent significant loci at *p* < 0.05. Selected candidate genes are labeled in the plots at their respective genomic positions

Furthermore, the allele frequencies of the annotated loci were associated with environmental gradients (Fig. 4a), indicating adaptation signals. Wind and soil gradients were significantly associated with the allele frequencies of *ATPD*, *NF-Y19*, *cob*, *rpoB*, and *ABCG34* (Fig. 4b; Table S7). Some outliers, such as *ADH1* orthologs, were associated with annual mean temperature and precipitation in spring (Fig. 4a). Other genes, including *AT2G40435*, the *NET1D* ortholog, and the *HSP70* ortholog, were also involved in precipitation- and temperature-associated adaptation (Fig. 4b; Table S7).

### Analyses of ecological niche and morphometric distinctiveness

Consistent with the genetic data, niche analysis of the first two axes of the environmental PCA and both overlap indices showed that the eastern and western clusters had a higher degree of niche overlap with each other than with HC (Fig. S7). HC was characterized by niches with higher mean annual temperature and lower precipitation in spring compared to those of the other clusters (Fig. S7). The equivalency test, background test, and ANOVA demonstrated that the three clusters occupied significantly different ecological niches (Fig. S7; Fig. S8).

**Fig. 5:**
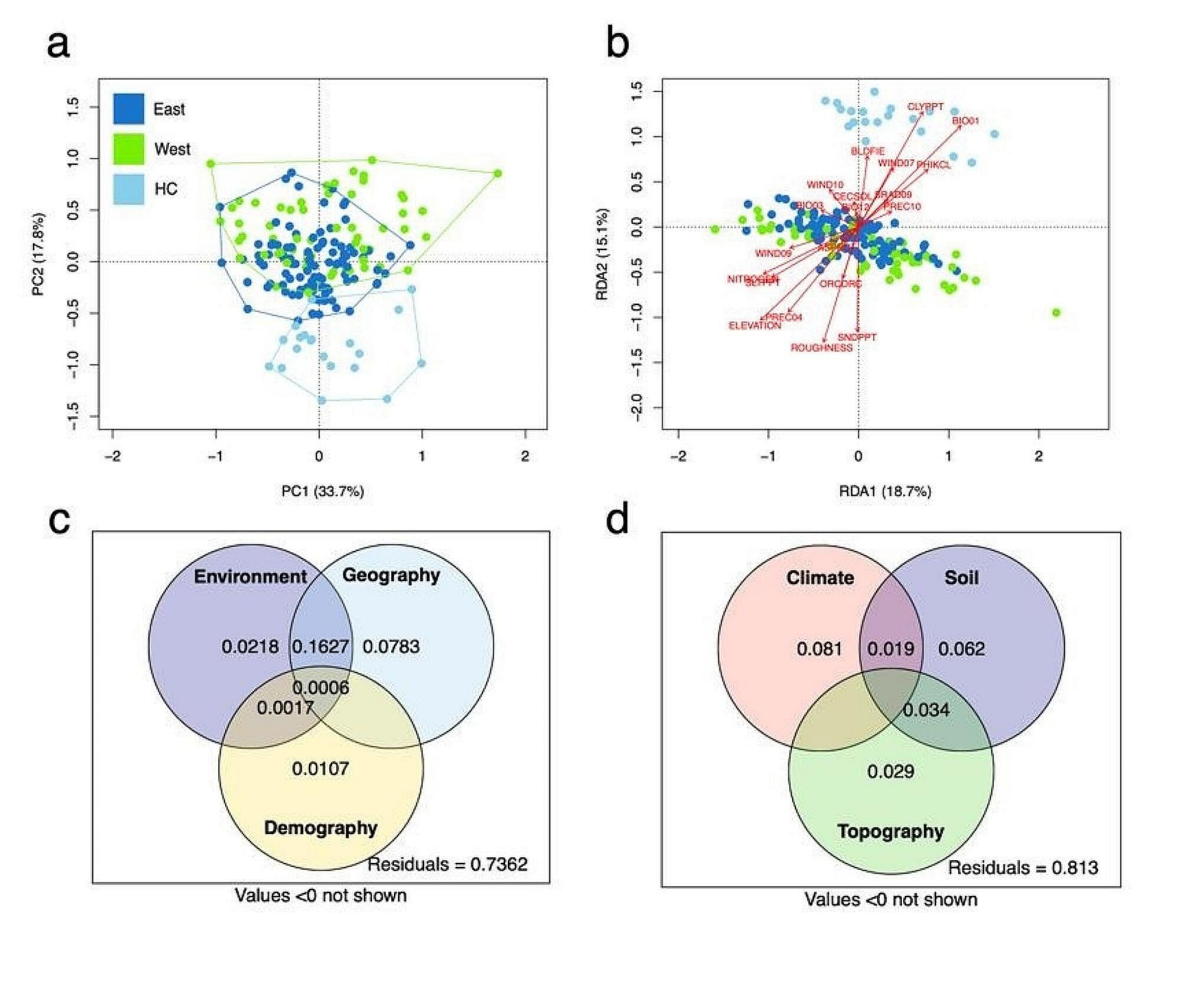
Variations in leaf traits and results of partial RDA. (a) Results of PCA colored by different genetic groups. (b) Partial RDA partitioning sources of phenotypic variation into different environmental factors. Environmental factors are depicted with red arrows. (c) Partial RDA of environmental factors, geography, and demography. (d) Partial RDA partitioning explained variation into climatic, soil, and topographical variables. The values are the explained variation. Values < 0 are not shown

The PCA and RDA of phenotypic traits were also congruent with the genetic analyses and showed that HC was morphologically and ecologically differentiated from the other clusters (Fig. 5a-b; Fig. S9; Fig. S10), which was classified as *Q. longinux* var. *kuoi*. Although the eastern and southern clusters had a high degree of overlap according to the first two PC axes, PERMANOVA indicated significant differentiation between the two clusters (Table S12). In contrast, *Q. longinux* var. *lativiolaciifolia* was genetically and morphologically admixed with *Q. longinux* var. *longinux*, and no apparent structure could be assigned between these two varieties (Fig. 1a-b). Partial RDA using all phenotypic traits as responses demonstrated that pure geography contributed more variation than pure environment, and a large intersection between their interactions was found (Fig. 5c-d; Table S13). The GLMs revealed that leaf traits were significantly associated with environment, but the directions of the relationships (i.e., positive or negative) differed depending on the trait and environmental variable (Fig. S11). For example, leaf thickness was positively correlated with annual temperature (*r* = 0.22, *p* < 0.01), whereas leaf length was negatively correlated with annual precipitation (*r* = − 0.22, *p* < 0.01).

### Genetic offset and prediction of the response to future climate change

**Fig. 6:**
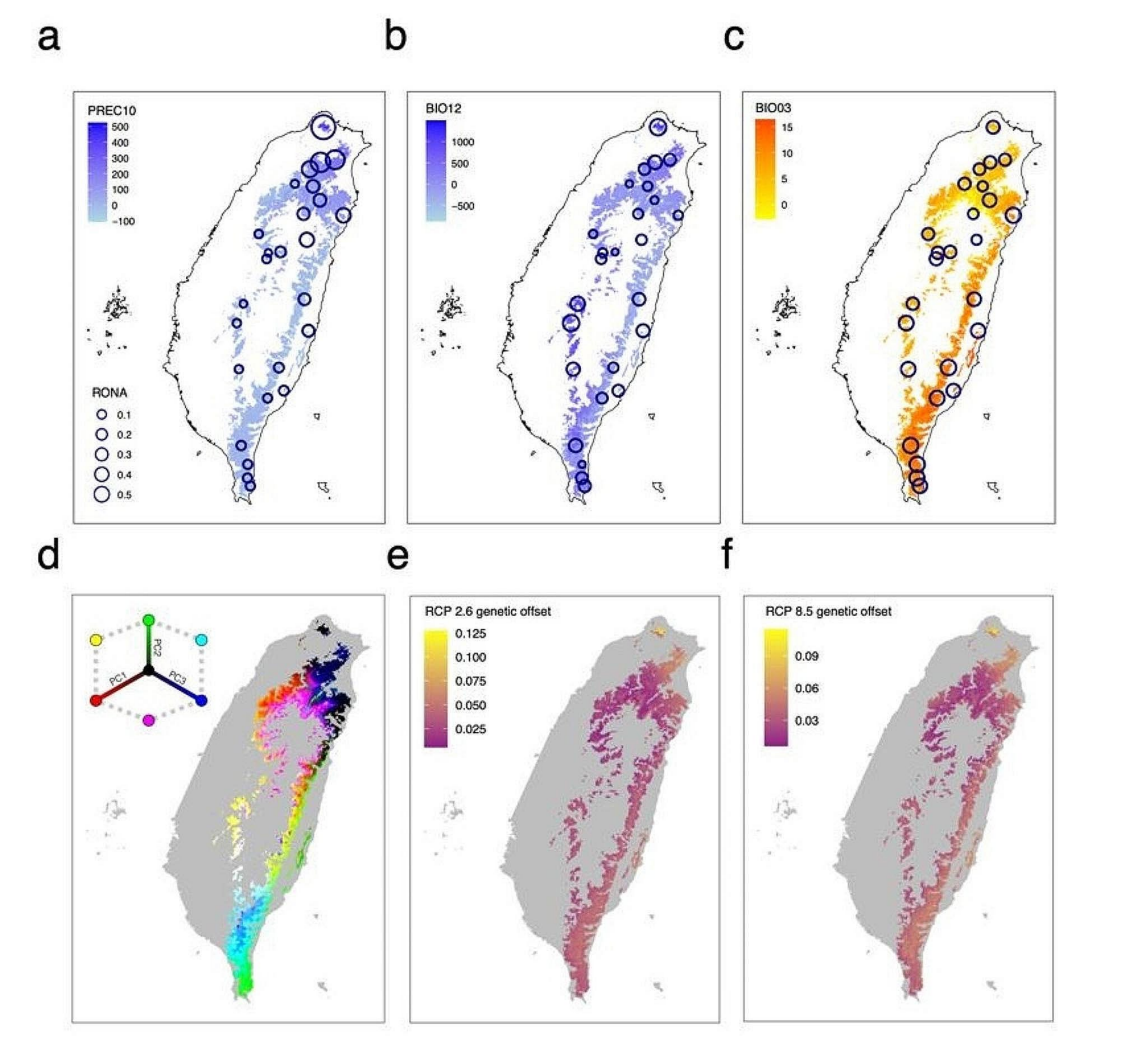
Prediction of genetic offset under future climate change based on (a, b, c) RONA and (d, e, f) GF methods. (a), (b), and (c) reflect the RONA values estimated for PREC10, BIO12, and BIO03. The color gradients represent the average differences in climatic variables between current conditions and 2070 climate change scenarios. (d) RGB map of the first three PC axes based on the GF prediction, which depicts the genetic turnover of adaptive loci. (e) and (f) illustrate the genetic offset throughout the range of *Q. longinux* under the RCP2.6 (c) and RCP8.5 (d) scenarios in 2070. The colors of the cells represent the values of genetic offset estimated based on the GF procedure

The high AUC value (average AUC = 0.81) suggested a good model fit for the predicted distribution of *Q. longinux* (Table S14; Fig. S12). As the predictions under different RCPs were highly correlated (Table S15), we inferred RONA based on the average values from both predictions. Substantial variations in the RONA estimates between populations and the top three representative environmental variables were observed (Fig. 6a-c; Table S15). The RONA estimates were larger in regions with greater differences between current and future climates (Fig. 6a-c). Whereas the eastern and western populations had relatively low RONA values (< 0.2) for the three variables, the northern populations were predicted to suffer from severe winter rainfall (precipitation in October, Fig. 6a) in the future and had much higher RONA values (> 0.6).

The GF model constructed with five climatic variables suggested that precipitation in winter was the most influential variable with the highest weighted *R*^2^ (Fig. S13). The focal species exhibited strong spatial patterns, indicating adaptation to local climate conditions (Fig. 6d). Consistent with the results of the RONA analysis, the GF model estimated highly correlated results between RCPs with similar genetic offset patterns (Fig. 6e-f). Both the RONA analysis and the GF model indicated that the northern populations were the most vulnerable to future climate change (Fig. 6e-f).

**Fig. 7:**
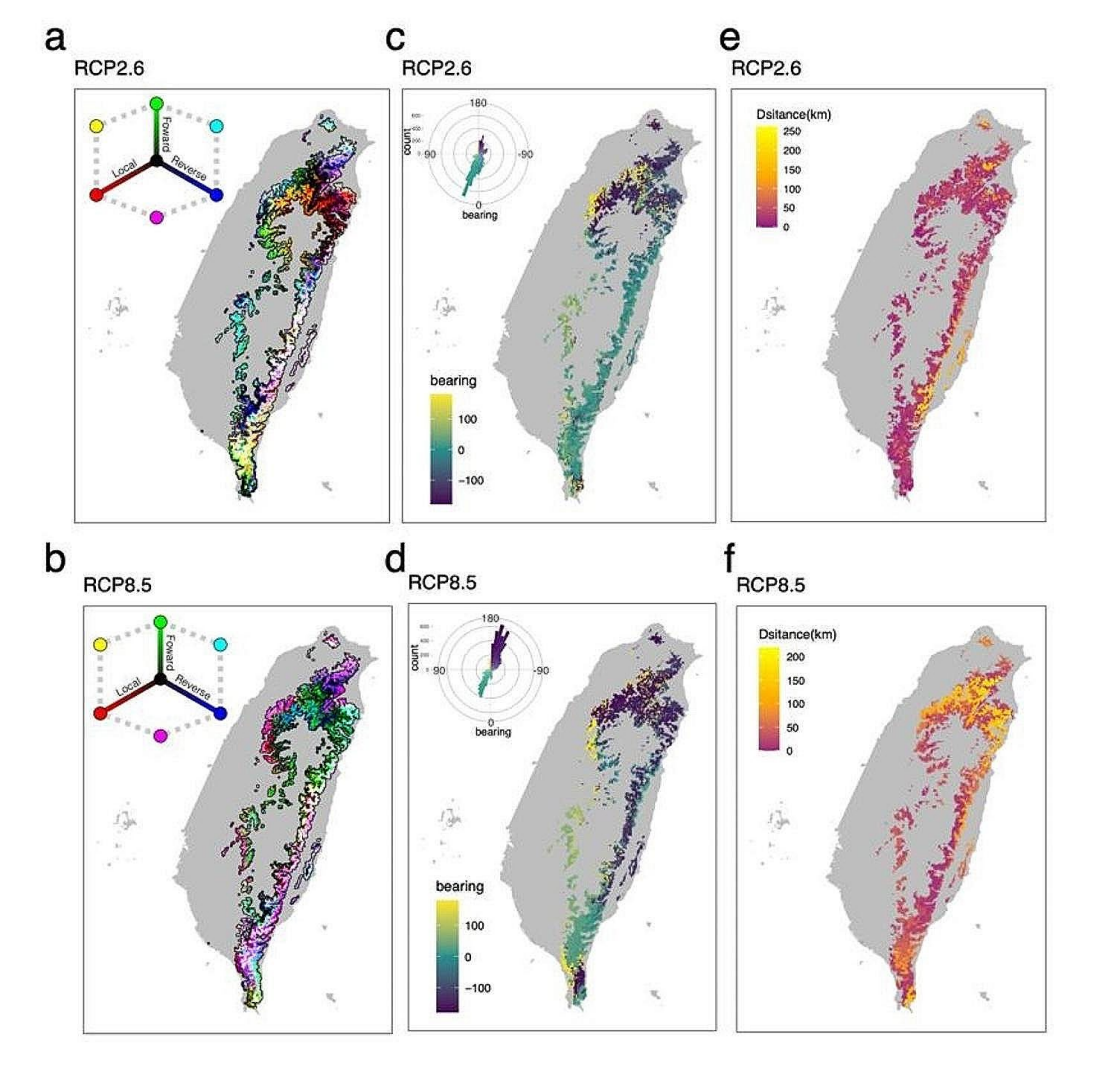
Map of the GDM-predicted genetic offset across the distribution of *Q. longinux* under the RCP2.6 (a, c, e) and RCP8.5 (b, d, f) climate change scenarios. (a) and (b) show the RGB maps of local (red), forward (green), and reverse (blue) offsets under RCP2.6 (a) and RCP8.5 (b) in 2070. Brighter cells represent relatively high values along each of the three axes, whereas darker cells indicate relatively low values. (c) and (d) depict the direction of forward offsets among the distribution range of *Q. longinux* under RCP2.6 (c) and RCP8.5 (d) in 2070. (e) and (f) represent the estimated distances between source cells with the lowest forward offset to sink cells under RCP2.6 (e) and RCP8.5 (f) in 2070

Although the predicted patterns of local, forward, and reverse offsets varied throughout the range of *Q. longinux*, these offsets were consistently predicted to be highest in the northernmost and southeastern populations (Fig. 7a-b). More migration events were predicted for the northern populations, with longer distances to minimize forward offsets in the RCP8.5 model than in the RCP2.6 model (Fig. 7c-f).

## Discussion

We analyzed genomic data and phenotypic traits to explore the genetic architecture of several environment-associated adaptations of *Q. longinux*, a dominant evergreen forest tree species on the subtropical island of Taiwan in East Asia. We identified several SNPs with strong effects on adaptation to environmental factors, including some factors that have rarely been discussed in GEA studies (e.g., soil- and wind-related factors). We found that leaf traits were influenced by the interaction of demographic and environmental factors. Moreover, we determined that the populations in northern and southeastern Taiwan are the most vulnerable to future climate change. Finally, we identified populations with unique genetic and phenotypic characteristics in southern Taiwan. These populations are potential targets for conservation efforts in forest management to preserve unique and adaptive genetic resources.

### Distinct genetic separation of southern populations from eastern and western populations in Taiwan

Three putative genetic clusters were classified using PCA and StrAuto. The eastern and western clusters were mainly separated from each other by the CMR and were mixed in northern and southern Taiwan. Similar patterns of east–west divergence have been observed for other plants in Taiwan [99, 100], implying that the mountain ridges and rugged topography act as profound barriers to gene flow and contribute to the divergence of species occupying low-to-middle elevations in Taiwan. The third cluster, HC, was limited to the Henchung Peninsula in southern Taiwan, which has been identified as the main glacial refuge in Taiwan for other Fagaceae species, such as *Q. glauca* and *Castanopsis carlesii* [101, 102]. The populations of HC were genetically differentiated from other populations, as suggested by higher pairwise *F*_ST_ and distinct trajectory of changes in *Ne* in the past 1,000 years. The optimal demographic scenario revealed HC diverged from the rest of the populations dating back to the Last Glacial Maximum (LGM; ∼20Kya) when most of the low-elevation oaks were believed to retreat to the refuge in southern Taiwan [101, 102]. Also, implying colonization resulting from Pleistocene climate oscillations could prompted the diversification and local adaptation of plants in Taiwan Island. Taken together, our results reveal significant genetic differentiation between the eastern cluster, western cluster, and HC and suggest that HC, morphologically classified as *Q. longinux* var. *kuoi*, in the southernmost part of Taiwan have diverged from the two clusters without apparent gene flow since their divergence.

A distinct group of *Q. longinux* populations, group HC, with unique leaf and fruit traits in tropical marine climates in southern Taiwan was previously classified as *Q. longinux* var. *kuoi*. This variety has no whitish epicuticular wax coating on abaxial surfaces [48]. Niche analyses also demonstrated that the habitats harboring *Q. longinux* var. *kuoi* were significantly different from those of the other populations, with no ecological overlaps, indicating that *Q. longinux* var. *kuoi* may face different environmental pressures. Moreover, we found evidence of adaptive divergence between *Q. longinux* var. *kuoi* and other populations. For example, the low spring precipitation and high clay content in habitats in southeastern Taiwan may act as strong environmental stresses that initiate genetic and phenotypic adaptation (e.g., drought resistance) in response to local conditions. In plant species, hostile environmental conditions in edge populations prompt local adaptation processes [103]. Distinct environmental pressure, along with a lack of migration between HC and the rest of the *Q. longinux* population, could speed up the fixation of alleles in a relatively smaller group, further facilitating the genetic and phenotypic adaptation [25, 104]. The substantial divergence, relatively high genetic diversity, and high offsets of the populations in southern Taiwan indicate that *Q. longinux* var. *kuoi* is a conservation unit that should be prioritized for protection as a source of adaptive genetic variations related to high temperature and drought resistance under climate change.

### Environmental heterogeneity drives adaptive genetic divergence and phenotypic variation

IBE was the most strongly supported model based on putative adaptive loci, whereas IBR mainly drove genetic differentiation based on all SNPs. PCA also revealed less genetic admixture when genetic differentiation was assessed by GEA outliers compared to neutral SNPs, indicating that the three genetic clusters (i.e., eastern, western, and HC) were exposed to different environmental pressures and had undergone adaptive divergence. However, the partial RDA revealed a large intersection of explained variation (45%) shared by environment, geography, and colonization history, suggesting that environmental variation is highly covaried with other confounding factors. Similar covariations have been observed in the south-to-north postglacial expansion of the red spruce along the Appalachian Mountains which created high collinearity between genetic structure, climate gradients, and geographic distributions [105]. Similarly, evergreen subtropical trees in Taiwan underwent south–north expansions after the LGM and may have developed adaptations to latitudinal gradients of temperature and precipitation, resulting in confounding relationships between geography, genetic structure, and adaptation. Consequently, it was challenging to attribute and disentangle the genetic variation explained by each category of predictors, leading to a non-significant impact of pure climate variables.

Leaf shape is affected by various environmental factors, such as precipitation and temperature, which maximize photosynthetic efficiency and adaptation to harsh environments [76, 106–108]. We found that several leaf traits of *Q. longinux* were influenced by environmental factors (Fig. S11; Table S13) and a negative correlation between leaf length and annual precipitation contradicts previous findings [51, 109]. However, we observed a positive correlation between annual precipitation and wind speed in winter and a negative correlation between annual precipitation and solar radiation in summer (Fig. S14). Strong wind and weaker solar radiation may counteract the effects of increased annual precipitation on leaf length. Similar confounding effects of climatic factors on leaf growth and elongation were observed in *Fagus sylvatica* [77]. We demonstrated that the interaction of environment and geographic relationships mainly contributed to the explained variation in leaf traits. Demographic history provided only a limited contribution to leaf variation, suggesting that phenotypic plasticity or local adaptation contributed by local climate surpasses the impact of demographic history on leaf traits.

Temperature and precipitation are known drivers of genetic adaptation in Fagaceae [34]. This study expands the environmental factors considered compared to previous GEA studies and demonstrates that significant selection is initiated by multiple environmental factors in an endemic *Quercus* species. First, we found that wind speed in cold seasons influenced leaf traits and adaptive genetic variation (Table S10; Fig. S11). Wind intensity has been shown to reduce plant growth and height and increase stem thickness [110, 111]. Wind also influences transpiration rates and stomatal conductance, indirectly affecting photosynthetic efficiency and water requirements [112, 113]. Some genes correlated with wind speed were also significantly associated with precipitation-related factors (e.g., *ATPD*, *NF-YCP*), implying that elevated evaporation rates caused by strong wind may result in drought-like stress, which plants may respond to through similar genetic pathways. Second, we determined that soil-related variables contributed 60% of the variation in adaptive divergence, indicating critical roles of these variables in local adaptation of *Q. longinux*. The characteristics of soil particle sizes represent the potential water content and salt stress in local soils. In general, soil particle size is negatively correlated with water availability, implying potential abiotic pressure from water deprivation during dry seasons [36, 114]. Consistent with this conclusion, we observed a correlation between genes involved in the response to drought and grid size. In summary, our findings provide a new perspective for future GEA studies by indicating that some environmental variables with important but rarely tested physiological impacts can be used to unravel the intricate mechanisms related to plant adaptation.

We identified two significantly enriched functional pathways, oxidative phosphorylation and photosynthesis, which have been implicated in plant adaptation and physiological responses to abiotic stress [115, 116]. Oxidative phosphorylation helps regulate reactive oxygen species (ROS) generation by plant mitochondria under abiotic stresses [115, 116]. The efficiency and regulation of photosynthesis strengthen the plant and sustain its growth and development under stressful or unfavorable conditions [117]. These results suggest that the identified adaptive SNPs underlie the response to different abiotic stresses. Several genes demonstrating significant associations with environments also have potential functions for adaptation. For example, loci of *ADH1* are strongly associated with annual mean temperature and precipitation in spring. *ADH1* is responsive to multiple abiotic stimuli, including low temperature, hypoxia, flooding, salt, and dehydration [118–120]. In legumes, *ADH1* is a target of miRNA regulation under water-deficit to coordinate ROS levels [121]. Under stressful cold situations, *ADH1* plays a crucial role in maintaining the stability of membrane structure to enhance cold resistance in plants [115]. Other genes involved in precipitation- and temperature-associated adaptation include orthologs of *AT2G40435*, which is involved in responses to biotic and abiotic stresses [122, 123]; *NET1D*, which is expressed in root tissues and mediates uptake in response to stress [124]; and *HSP70*, which stabilizes and refolds heat-inactivated proteins to protect cells from heat damage [125].

### Assessing genetic vulnerability and climate adaptation in *Quercus longinux*

The projections of genetic offset from GF and RONA revealed that the populations in northern Taiwan might experience the most considerable turnover of genetic composition to cope with future climate change (Fig. 6). Winter precipitation in northern Taiwan is expected to more than double according to both emission models (current: 302 mm, RCP2.6: 682 mm, RCP8.5: 635 mm). The drastic increase in winter precipitation will negatively impact forest productivity [126] because the complex relationship between precipitation and water availability affects plant growth and phenology [126, 127]. Winter precipitation significantly impacts the phenology of oaks, including the onset and duration of flowering, bud bursting, and leaf flushing, and thus may greatly affect the likelihood and extent of gene flow between populations [128]. Considering the long generation time of oaks and the difficulty of juvenile growth in occupied forests, the expected changes in allele frequency to adapt to increased winter precipitation in the northern populations (RONA > 0.6) may not be achievable through standing genetic variation alone.

Under the emission model of intensified global warming (RCP8.5), southward migration over long distances (> 200 km) will increase to minimize forward genetic offset. Although we accounted for migration in estimating genetic offset, the northernmost and southeastern populations consistently showed relatively high local, forward, and reverse offsets. Our results indicate that no current populations across the distribution of *Q. longinux* are preadapted to future climates in these regions. The northern and southeastern populations are crucial genetic sources for climatic adaptation in other regions and should be prioritized in conservation strategies and protection efforts. Moreover, the rugged topography in mountainous regions may further hamper the movement of populations to higher elevations. Assisted gene flow from other populations preadapted to future climates may help marginal populations mitigate the effects of climate change [129]. For example, southern populations (e.g., HC) may act as potential sources of adaptation to high temperatures and seasonal arid climates for populations at higher altitudes and latitudes where higher future temperatures are predicted. However, the source populations must be selected carefully to ensure genetic compatibility with the sink populations and new environments [129].

### Electronic supplementary material

Below is the link to the electronic supplementary material.

Supplementary Material 1

Supplementary Material 2

## Data Availability

Sequencing data have been deposited on NCBI BioProject PRJNA1049506 under GenBank accession number SAMN38699773–SAMN38700060. The Variant Call Format (VCF) file along with additional files used for the analyses have been deposited on Figshare under DOI 10.6084/m9.figshare.24276946.

## References

[1] Aitken SN (2008). Adaptation, migration or extirpation: climate change outcomes for tree populations. Evol Appl.

[2] Fitzpatrick MC, Keller SR (2015). Ecological genomics meets community-level modelling of biodiversity: mapping the genomic landscape of current and future environmental adaptation. Ecol Lett.

[3] Rellstab C, Dauphin B, Exposito-Alonso M (2021). Prospects and limitations of genomic offset in conservation management. Evol Appl.

[4] Seidl R (2017). Forest disturbances under climate change. Nat Clim Change.

[5] Kramer K, Leinonen I, Loustau D (2000). The importance of phenology for the evaluation of impact of climate change on growth of boreal, temperate and Mediterranean forests ecosystems: an overview. Int J Biometeorol.

[6] Piao S (2019). Plant phenology and global climate change: current progresses and challenges. Glob Change Biol.

[7] Flannigan MD, Stocks BJ, Wotton BM (2000). Climate change and forest fires. Sci Total Environ.

[8] Kirschbaum MU, Fischlin A. *Climate change impacts on forests* 1996.

[9] Lindner M (2010). Climate change impacts, adaptive capacity, and vulnerability of European forest ecosystems. For Ecol Manag.

[10] Way DA, Oren R (2010). Differential responses to changes in growth temperature between trees from different functional groups and biomes: a review and synthesis of data. Tree Physiol.

[11] Wang T, O’Neill GA, Aitken SN (2010). Integrating environmental and genetic effects to predict responses of tree populations to climate. Ecol Appl.

[12] Pedlar JH, McKenney DW (2017). Assessing the anticipated growth response of northern conifer populations to a warming climate. Sci Rep.

[13] Saxe H (2001). Tree and forest functioning in response to global warming. New Phytol.

[14] Waring RH, Schlesinger W. *Forest ecosystems* Analysis at multiples scales, 1985. 55.

[15] Camarero JJ (2013). Growth response to climate and drought change along an aridity gradient in the southernmost Pinus nigra relict forests. Ann for Sci.

[16] Lines ER (2012). Predictable changes in aboveground allometry of trees along gradients of temperature, aridity and competition. Glob Ecol Biogeogr.

[17] Waring RH (1987). Characteristics of trees predisposed to die. Bioscience.

[18] Ramírez-Valiente JA, Cavender-Bares J (2017). Evolutionary trade-offs between drought resistance mechanisms across a precipitation gradient in a seasonally dry tropical oak (Quercus oleoides). Tree Physiol.

[19] Sork VL (2018). Genomic studies of local adaptation in natural plant populations. J Hered.

[20] Waldvogel A-M (2020). Climate change genomics calls for standardized data reporting. Front Ecol Evol.

[21] Rellstab C (2015). A practical guide to environmental association analysis in landscape genomics. Mol Ecol.

[22] Gugger PF (2021). Landscape genomics of Quercus lobata reveals genes involved in local climate adaptation at multiple spatial scales. Mol Ecol.

[23] Cavender-Bares J (2019). Diversification, adaptation, and community assembly of the American oaks (Quercus), a model clade for integrating ecology and evolution. New Phytol.

[24] Gao J (2021). Combined genotype and phenotype analyses reveal patterns of genomic adaptation to local environments in the subtropical oak Quercus acutissima. J Syst Evol.

[25] Sork V (2013). Putting the landscape into the genomics of trees: approaches for understanding local adaptation and population responses to changing climate. Tree Genet Genomes.

[26] Holderegger R (2010). Landscape genetics of plants. Trends Plant Sci.

[27] Richardson JL, et al. Navigating the pitfalls and promise of landscape genetics. Wiley Online Library; 2016. 10.1111/mec.13527 26756865

[28] Razgour O (2015). Beyond species distribution modeling: a landscape genetics approach to investigating range shifts under future climate change. Ecol Inf.

[29] Razgour O (2019). Considering adaptive genetic variation in climate change vulnerability assessment reduces species range loss projections. Proc Natl Acad Sci.

[30] Pollegioni P (2014). Landscape genetics of persian walnut (Juglans regia L.) across its Asian range. Tree Genet Genomes.

[31] Mattioni C (2017). Landscape genetics structure of European sweet chestnut (Castanea sativa Mill): indications for conservation priorities. Tree Genet Genomes.

[32] Petit RJ (2013). Fagaceae trees as models to integrate ecology, evolution and genomics. New Phytol.

[33] Kim BY (2018). RADseq data reveal ancient, but not pervasive, introgression between Californian tree and scrub oak species (Quercus sect. Quercus: Fagaceae). Mol Ecol.

[34] Müller M, Gailing O (2019). Abiotic genetic adaptation in the Fagaceae. Plant Biol.

[35] Aguirre-Liguori JA (2019). Divergence with gene flow is driven by local adaptation to temperature and soil phosphorus concentration in teosinte subspecies (Zea mays parviglumis and Zea mays mexicana). Mol Ecol.

[36] Rellstab C (2016). Signatures of local adaptation in candidate genes of oaks (Quercus spp.) with respect to present and future climatic conditions. Mol Ecol.

[37] Macel M (2007). Climate vs. soil factors in local adaptation of two common plant species. Ecology.

[38] Cavender-Bares J, Ramírez-Valiente JA. Physiological evidence from common garden experiments for local adaptation and adaptive plasticity to climate in American live oaks (Quercus Section Virentes): implications for conservation under global change. Oaks physiological ecology. Exploring the functional diversity of genus Quercus L. Springer; 2017. pp. 107–35.

[39] Smith DS (2012). Soil-mediated local adaptation alters seedling survival and performance. Plant Soil.

[40] Byars SG, Papst W, Hoffmann AA (2007). Local adaptation and cogradient selection in the alpine plant, Poa Hiemata, along a narrow altitudinal gradient. Evolution: Int J Org Evol.

[41] Fang J-Y (2013). Divergent selection and local adaptation in disjunct populations of an endangered conifer, Keteleeria davidiana var. Formosana (Pinaceae). PLoS ONE.

[42] Hsieh Y (2013). Historical connectivity, contemporary isolation and local adaptation in a widespread but discontinuously distributed species endemic to Taiwan, Rhododendron Oldhamii (Ericaceae). Heredity.

[43] Huang C-L (2015). Genetic relationships and ecological divergence in Salix species and populations in Taiwan. Tree Genet Genomes.

[44] Foster P (2001). The potential negative impacts of global climate change on tropical montane cloud forests. Earth Sci Rev.

[45] Dirnböck T, Essl F, Rabitsch W (2011). Disproportional risk for habitat loss of high-altitude endemic species under climate change. Glob Change Biol.

[46] Still CJ, Foster PN, Schneider SH (1999). Simulating the effects of climate change on tropical montane cloud forests. Nature.

[47] Cazzolla Gatti R (2019). Accelerating upward treeline shift in the Altai Mountains under last-century climate change. Sci Rep.

[48] Huang T. *Flora of Taiwan, 2nd edn, Vols 1–5* Editorial Committee of the Flora of Taiwan, Taipei, 1994.

[49] Zhong M (2014). Leaf morphology shift of three dominant species along altitudinal gradient in an alpine meadow of the Qinghai-Tibetan Plateau. Pol J Ecol.

[50] Vaca-Sánchez MS (2021). Genetic and functional leaf traits variability of Quercus Laurina along an oak diversity gradient in Mexico. Eur J for Res.

[51] Peppe DJ (2011). Sensitivity of leaf size and shape to climate: global patterns and paleoclimatic applications. New Phytol.

[52] Doyle J. *DNA protocols for plants*, in *Molecular techniques in Taxonomy*. Springer; 1991. pp. 283–93.

[53] Chen S (2018). Fastp: an ultra-fast all-in-one FASTQ preprocessor. Bioinformatics.

[54] Schmid-Siegert E (2017). Low number of fixed somatic mutations in a long-lived oak tree. Nat Plants.

[55] Li H. *Aligning sequence reads, clone sequences and assembly contigs with BWA-MEM* arXiv preprint arXiv:1303.3997, 2013.

[56] Mose LE, Perou CM, Parker JS (2019). Improved indel detection in DNA and RNA via realignment with ABRA2. Bioinformatics.

[57] Li H (2009). The sequence alignment/map format and SAMtools. Bioinformatics.

[58] Li H (2011). A statistical framework for SNP calling, mutation discovery, association mapping and population genetical parameter estimation from sequencing data. Bioinformatics.

[59] Danecek P (2011). The variant call format and VCFtools. Bioinformatics.

[60] Jombart T (2008). Adegenet: a R package for the multivariate analysis of genetic markers. Bioinformatics.

[61] Goudet J (2005). Hierfstat, a package for R to compute and test hierarchical F-statistics. Mol Ecol Notes.

[62] Oksanen J (2013). *Package ‘vegan’* community ecology package. Version.

[63] Paradis E, Claude J, Strimmer K (2004). APE: analyses of phylogenetics and evolution in R language. Bioinformatics.

[64] Chhatre VE, Emerson KJ (2017). StrAuto: automation and parallelization of STRUCTURE analysis. BMC Bioinformatics.

[65] Kopelman NM (2015). Clumpak: a program for identifying clustering modes and packaging population structure inferences across K. Mol Ecol Resour.

[66] Liu X, Fu Y-X (2020). Stairway plot 2: demographic history inference with folded SNP frequency spectra. Genome Biol.

[67] Kleinschmit J. *Intraspecific variation of growth and adaptive traits in European oak species*. In *Annales des sciences forestières*. EDP Sciences; 1993.

[68] Excoffier L (2021). fastsimcoal2: demographic inference under complex evolutionary scenarios. Bioinformatics.

[69] Fick SE, Hijmans RJ (2017). WorldClim 2: new 1-km spatial resolution climate surfaces for global land areas. Int J Climatol.

[70] Trabucco A, Zomer RJ. Global aridity index and potential evapotranspiration (ET0) climate database v2. CGIAR Consort Spat Inf; 2018. p. 10.

[71] Poggio L (2021). SoilGrids 2.0: producing soil information for the globe with quantified spatial uncertainty. Soil.

[72] Hijmans RJ et al. *Raster package in R*. 2013, Version.

[73] Core Team R. R., *R: A language and environment for statistical computing* 2013.

[74] Di Cola V (2017). Ecospat: an R package to support spatial analyses and modeling of species niches and distributions. Ecography.

[75] Warren DL, Glor RE, Turelli M (2010). ENMTools: a toolbox for comparative studies of environmental niche models. Ecography.

[76] Sun M (2016). Variations in leaf morphological traits of Quercus guyavifolia (Fagaceae) were mainly influenced by water and ultraviolet irradiation at high elevations on the Qinghai-Tibet Plateau, China. Int J Agric Biol.

[77] Meier IC, Leuschner C (2008). Leaf size and leaf area index in Fagus sylvatica forests: competing effects of precipitation, temperature, and nitrogen availability. Ecosystems.

[78] Kremer A (2002). Leaf morphological differentiation between Quercus robur and Quercus petraea is stable across western European mixed oak stands. Ann for Sci.

[79] Abràmoff MD, Magalhães PJ, Ram SJ (2004). Image processing with ImageJ. Biophotonics Int.

[80] Kassambara A, Mundt F. Package ‘factoextra’. Extract Visualize Results Multivar data Analyses, 2017. 76(2).

[81] Ripley B (2013). Package ‘mass’. Cran r.

[82] Foll M. BayeScan v2. 1 user manual. Ecology, 2012. 20(10).

[83] Luu K, Bazin E, Blum MG (2017). Pcadapt: an R package to perform genome scans for selection based on principal component analysis. Mol Ecol Resour.

[84] Thissen D, Steinberg L, Kuang D (2002). Quick and easy implementation of the Benjamini-Hochberg procedure for controlling the false positive rate in multiple comparisons. J Educational Behav Stat.

[85] Frichot E, François O (2015). LEA: an R package for landscape and ecological association studies. Methods Ecol Evol.

[86] Frichot E (2013). Testing for associations between loci and environmental gradients using latent factor mixed models. Mol Biol Evol.

[87] Pitcher C et al. *Example analysis of biodiversity survey data with R package gradientForest* R vignette. Available at http://gradientforest. r-forge. r-project. org/biodiversity-survey. pdf [Verified 27 March 2017], 2011.

[88] Neph S (2012). BEDOPS: high-performance genomic feature operations. Bioinformatics.

[89] Bu D (2021). KOBAS-i: intelligent prioritization and exploratory visualization of biological functions for gene enrichment analysis. Nucleic Acids Res.

[90] Upton GJ (1992). Fisher’s exact test. J Royal Stat Society: Ser (Statistics Society).

[91] McRae BH, Shah VB (2009). Circuitscape user’s guide.

[92] Cushman SA (2013). Re-evaluating causal modeling with mantel tests in landscape genetics. Diversity.

[93] Peterman WE (2019). A comparison of popular approaches to optimize landscape resistance surfaces. Landscape Ecol.

[94] Peterman WE (2018). ResistanceGA: an R package for the optimization of resistance surfaces using genetic algorithms. Methods Ecol Evol.

[95] Petkova D, Novembre J, Stephens M (2016). Visualizing spatial population structure with estimated effective migration surfaces. Nat Genet.

[96] Naimi B, Araújo MB (2016). Sdm: a reproducible and extensible R platform for species distribution modelling. Ecography.

[97] Pina-Martins F (2019). New insights into adaptation and population structure of cork oak using genotyping by sequencing. Glob Change Biol.

[98] Gougherty AV, Keller SR, Fitzpatrick MC (2021). Maladaptation, migration and extirpation fuel climate change risk in a forest tree species. Nat Clim Change.

[99] Yu T-L, Lin H-D, Weng C-F (2014). A new phylogeographic pattern of endemic Bufo bankorensis in Taiwan Island is attributed to the genetic variation of populations. PLoS ONE.

[100] Huang SF (2004). Phylogeography of Trochodendron aralioides (Trochodendraceae) in Taiwan and its adjacent areas. J Biogeogr.

[101] Shih F (2006). Partial concordance between nuclear and organelle DNA in revealing the genetic divergence among Quercus glauca (Fagaceae) populations in Taiwan. Int J Plant Sci.

[102] Cheng YP, Hwang SY, Lin TP (2005). Potential refugia in Taiwan revealed by the phylogeographical study of Castanopsis Carlesii Hayata (Fagaceae). Mol Ecol.

[103] Yang Y-Z (2022). Parallel adaptation prompted core-periphery divergence of Ammopiptanthus mongolicus. Front Plant Sci.

[104] Manel S (2003). Landscape genetics: combining landscape ecology and population genetics. Trends Ecol Evol.

[105] Capblancq T (2023). From common gardens to candidate genes: exploring local adaptation to climate in red spruce. New Phytol.

[106] Joel G, Aplet G, Vitousek PM. *Leaf morphology along environmental gradients in Hawaiian Metrosideros polymorpha* Biotropica, 1994: p. 17–22.

[107] Liu W, Zheng L, Qi D (2020). Variation in leaf traits at different altitudes reflects the adaptive strategy of plants to environmental changes. Ecol Evol.

[108] Hovenden MJ, Vander JK, Schoor (2004). Nature vs nurture in the leaf morphology of Southern Beech, Nothofagus Cunninghamii (Nothofagaceae). New Phytol.

[109] Wiemann MC (1998). Estimation of temperature and precipitation from morphological characters of dicotyledonous leaves. Am J Bot.

[110] Biddington NL (1986). The effects of mechanically-induced stress in plants—a review. Plant Growth Regul.

[111] Smith V, Ennos A (2003). The effects of air flow and stem flexure on the mechanical and hydraulic properties of the stems of sunflowers Helianthus annuus L. J Exp Bot.

[112] Anten NP (2010). Wind and mechanical stimuli differentially affect leaf traits in Plantago major. New Phytol.

[113] Onoda Y, Anten NP (2011). Challenges to understand plant responses to wind. Plant Signal Behav.

[114] Zhang X (2019). Relationship between soil water content and soil particle size on typical slopes of the Loess Plateau during a drought year. Sci Total Environ.

[115] Zsigmond L (2008). Arabidopsis PPR40 connects abiotic stress responses to mitochondrial electron transport. Plant Physiol.

[116] Vashisth D (2018). Transcriptome changes induced by abiotic stresses in Artemisia annua. Sci Rep.

[117] Muhammad I (2021). Mechanisms regulating the dynamics of photosynthesis under abiotic stresses. Front Plant Sci.

[118] Christie PJ, Hahn M, Walbot V (1991). Low-temperature accumulation of alcohol dehydrogenase-1 mRNA and protein activity in maize and rice seedlings. Plant Physiol.

[119] de Bruxelles GL (1996). Abscisic acid induces the alcohol dehydrogenase gene in Arabidopsis. Plant Physiol.

[120] Komatsu S (2011). Characterization of a novel flooding stress-responsive alcohol dehydrogenase expressed in soybean roots. Plant Mol Biol.

[121] De la Rosa C, Covarrubias AA, Reyes JL (2019). A dicistronic precursor encoding miR398 and the legume-specific miR2119 coregulates CSD1 and ADH1 mRNAs in response to water deficit. Plant Cell Environ.

[122] Liu JX (2007). Salt stress responses in Arabidopsis utilize a signal transduction pathway related to endoplasmic reticulum stress signaling. Plant J.

[123] Lin F (2014). Molecular response to the pathogen Phytophthora sojae among ten soybean near isogenic lines revealed by comparative transcriptomics. BMC Genomics.

[124] Hawkins TJ (2014). The evolution of the actin binding NET superfamily. Front Plant Sci.

[125] De Maio A (1999). Heat shock proteins: facts, thoughts, and dreams. Shock.

[126] Zeppel M, Wilks JV, Lewis JD (2014). Impacts of extreme precipitation and seasonal changes in precipitation on plants. Biogeosciences.

[127] Lipton D, et al. Ecosystems, ecosystem services, and biodiversity. US Global Change Research Program; 2018.

[128] Armstrong-Herniman W, Greenwood S (2021). The role of winter precipitation as a climatic driver of the spring phenology of five California Quercus species (Fagaceae). Madroño.

[129] Aitken SN, Whitlock MC (2013). Assisted gene flow to facilitate local adaptation to climate change. Annu Rev Ecol Evol Syst.

