# Supplementary material for: Genomic insights into local adaptation and vulnerability of Quercus longinux to climate change
Source: BMC Plant Biol. 2024 Apr 13;24:279. doi: 10.1186/s12870-024-04942-8 (PMC11015620; doi:10.1186/s12870-024-04942-8)
Supplement: Supplementary file 1 — Supplementary Material 1 [file 12870_2024_4942_MOESM1_ESM.docx]

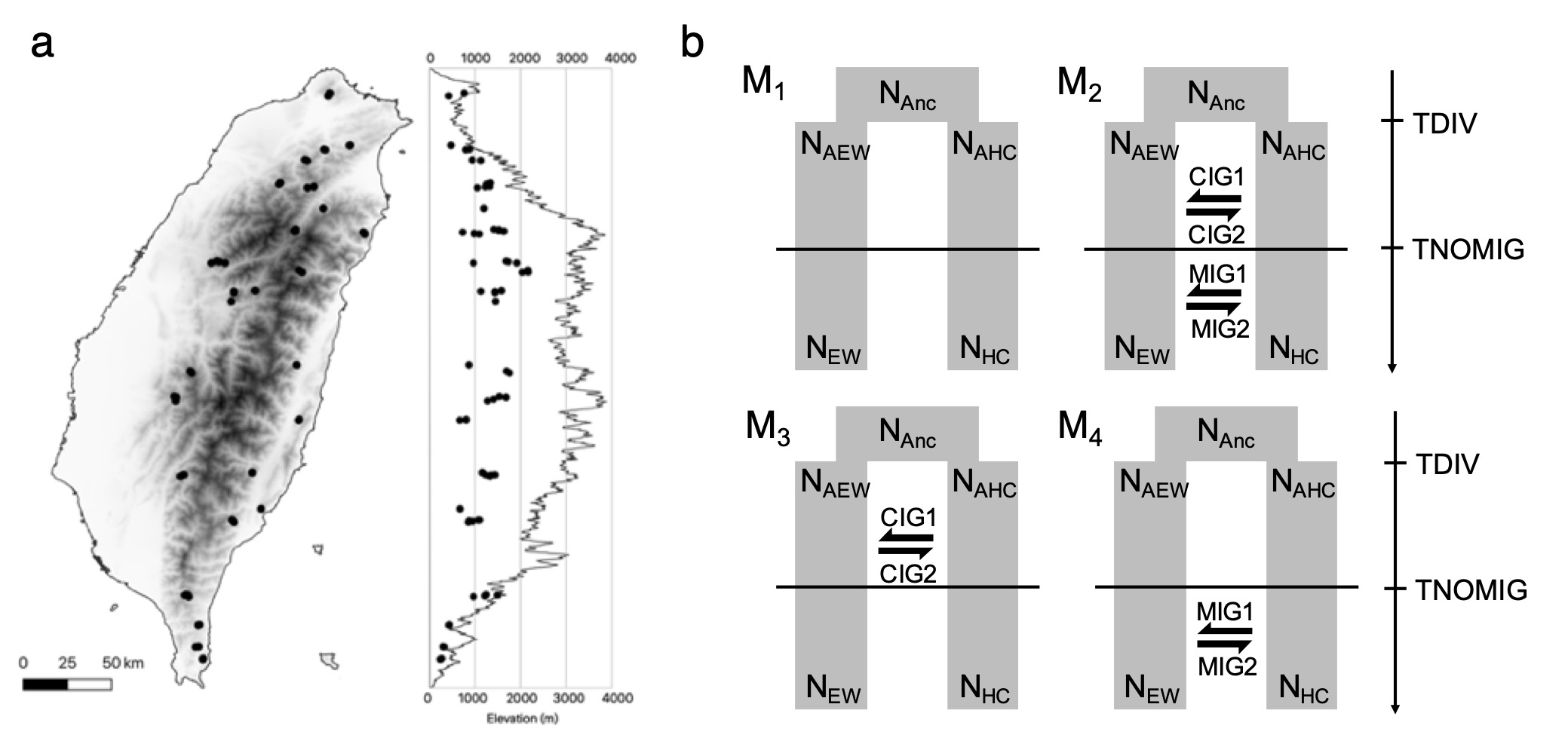


Fig. S1 Sampling sites in Taiwan and simulated evolution scenarios. (a) sampling individuals. Sites were represented by black dots. (b) Simulated demographic models. M1, model without migration. M2, model of continuous migration. M3, model of early migration. M4, model fo secondary contact. NEW, current population size of East and West group; NHC, current population size of HC group; TDIV, divergence time between EW and HC; TNOMIG, time with changes in population sizes or migration rates; NAEW, population size of East and West group before TNOMIG; NAHC, population size of HC group before TNOMIG; NANC, population size of the ancestral population; MIG1, current migration rate from HC to EW; MIG2, current migration rate from EW to HC; CIG1, migration rate from HC to EW before TNOMIG; CIG2, migration rate from EW to HC before TNOMIG.


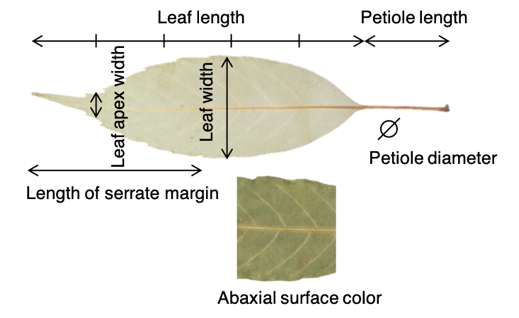


Fig. S2 Diagram showing the measured leaf traits. Descriptions of each trait are provided in Table S4.

Fig. S3 Genetic differentiation assessed with (a) population-specific F_ST_ and (b) Neighbor-joining tree. Colors represent the values of population-specific F_ST_.


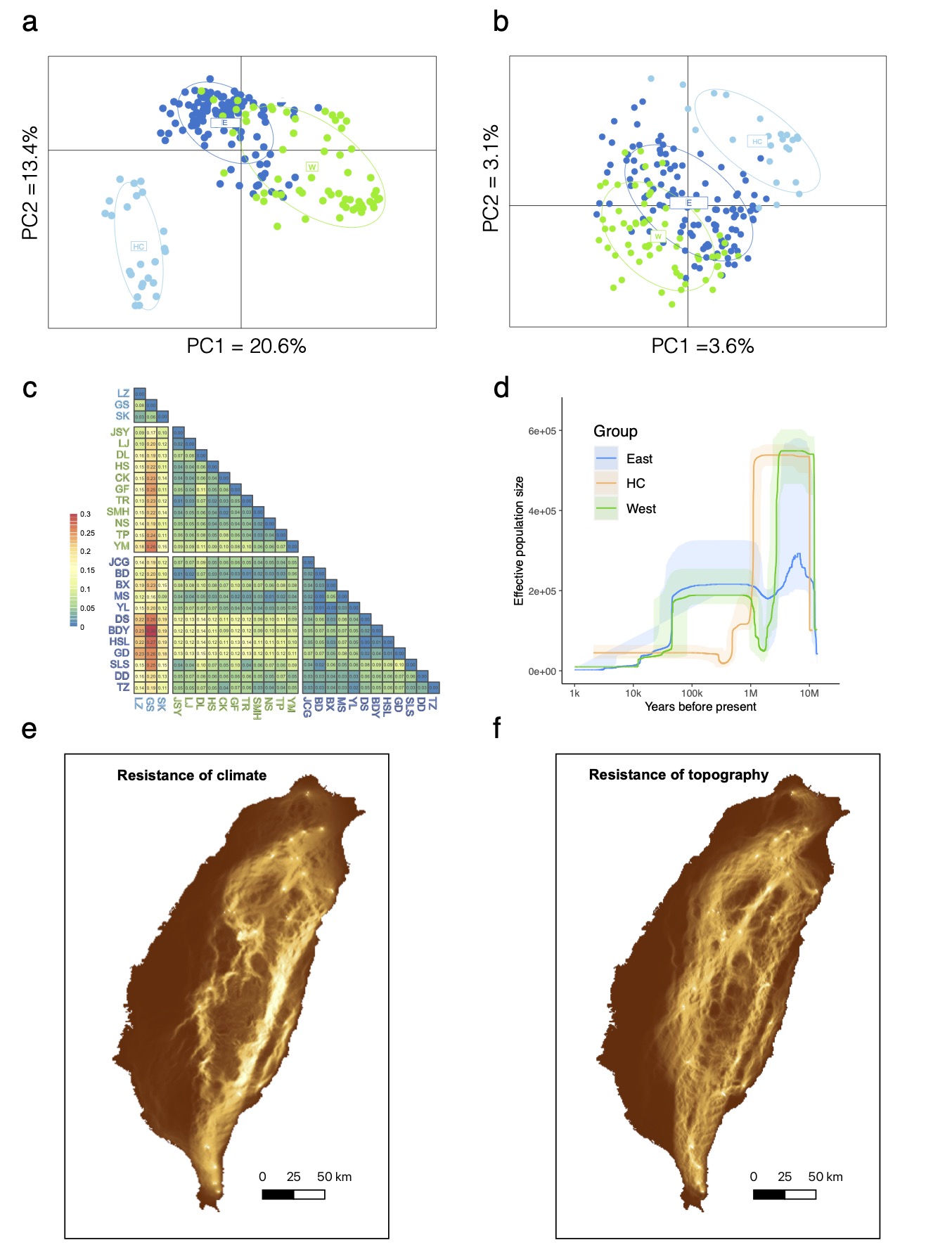


Fig. S4 Genetic structures, demographic history, and resistance maps. Results of PCA based on (a) adaptive loci and (b) neutral loci. (c) pairwise FST between populations (n = 26). Population names are colored by assigned genetic groups. (d) Inference of the demographic history of different groups of *Q. longinux*. Solid lines represent the mean population size (*Ne*) estimated by the Stairway Plot method. Semi-transparent colors represent 95% CI of Ne for each group. (e) Potential corridors for gene flow in the isolation by resistance (IBR) models constructed using climatic factors and (f) topographical factors. Lighter colors represent greater ease of movement (conductance) through the landscape.


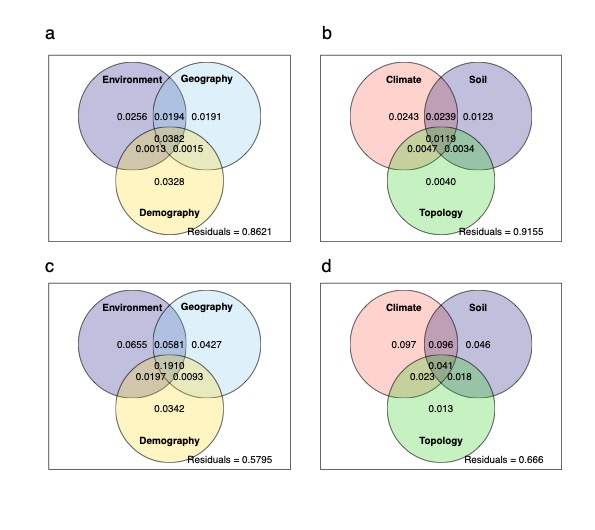


Fig. S5 Results of partial RDA based on all SNP sites (a, b) and adaptive loci (c, d). Partial RDA partitioning sources of genetic variation of all SNP sites into (a) variables of environments, geography, and demography. (b) Partial RDA dissecting variation explained by each of three categories of environmental variables (i.e., climatic, soil, and topographical variables). (c) Partial RDA performed with adaptive loci dividing contribution brought by environments, geography, and demography. (d) Partial RDA partitioned explained variation into climatic, soil, and topographical variables based on adaptive loci.

Fig. S6 Results of the GF model performed with variables of environment, geography, and demography based on adaptive outliers. (a) Genetic turnover along each variable. (b) Accuracy and R^2^-weighted importance of all variables.


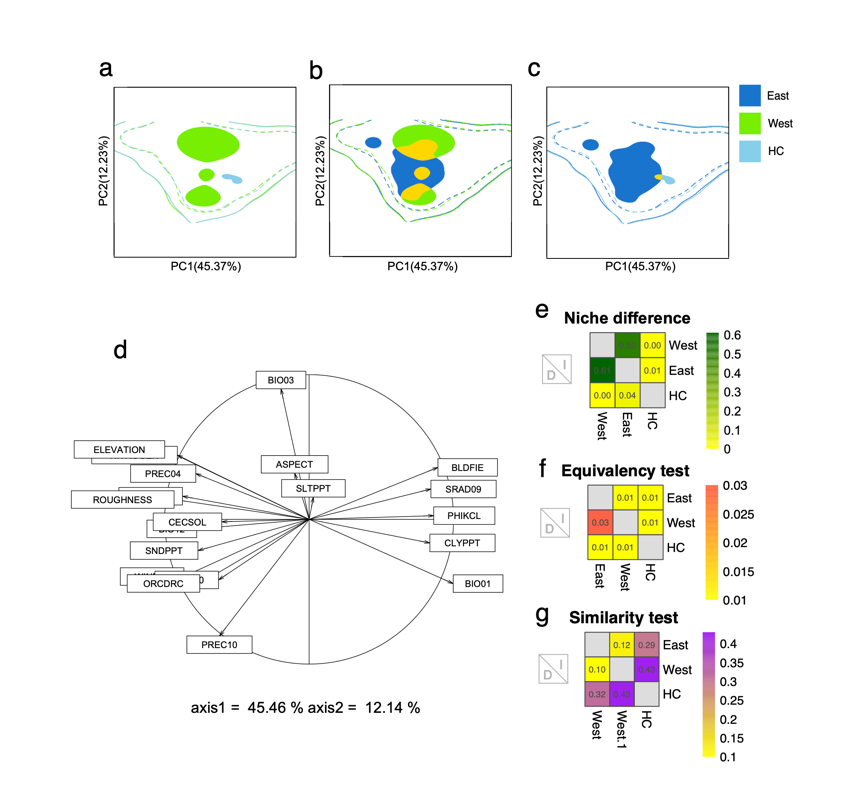


Fig. S7 Niches of the three genetic groups (i.e., Easter, Wester, and HC) in the environmental space of the study area. Niche overlaps are depicted between (a) the Western group and HC; (b) the Eastern and Western groups; and (c) the Eastern group and HC. (d) The contribution of the environmental variables to the two axes of the environmental PCA and the percentage of variation explained by the two axes. (e) Values of niche overlap between groups. Colors represent the overlap of niches from 0 (no overlap) to 1 (complete overlap). Niche differentiation was tested using (f) the equivalency test and (g) the similarity test. Colors represent the p-values of each test. Each test was run with Hellinger ’I (upper triangular) and Schoener’ D (lower triangular) separately.

Fig. S8 Comparison of obtained environmental variables between groups with ANOVA. Groups labeled with different colors represent significantly different values of mean.

Fig. S9 Comparison of leaf traits between groups with ANOVA. Groups labeled with different colors represent significantly different values of mean. Full names and descriptions of each trait are presented in Table 4.

Fig. S10 Biplot of PCA based on leaf traits. The colors of variables indicate the contribution assessed from the first two PC axes.

Fig. S11 Associations between environmental variables and leaf traits assessed by univariable GLMs. (a) P-value of each model. Orange cells indicate a significant correlation (P < 0.05). (b) Pearson correlation coefficient (r) between variables.


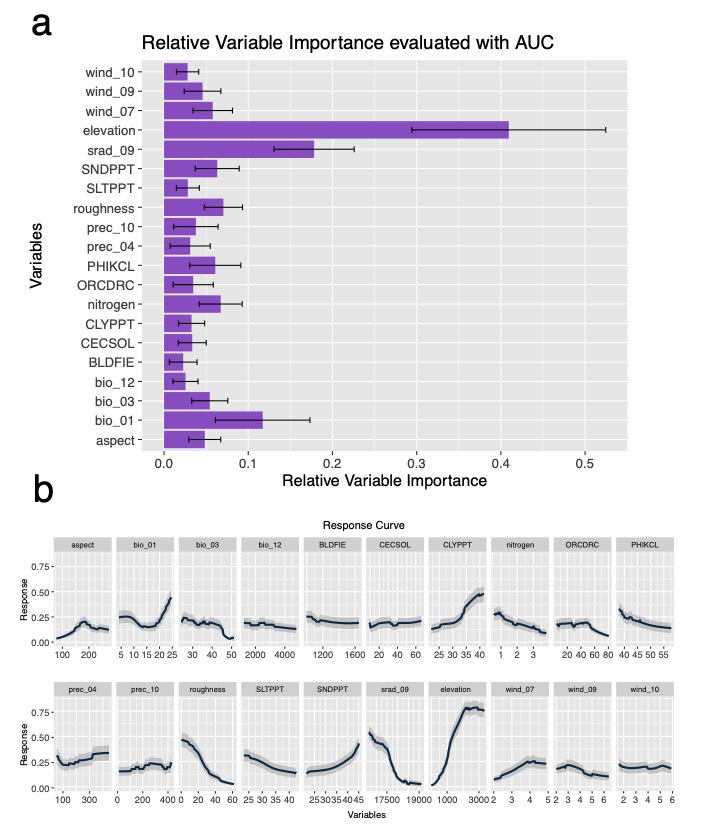


Fig. S12 Results of ENM constructed with ensemble approach. (a) Relative importance of each variable. (b) Response curve of each variable.

Fig. S13 Results of the GF model performed with climatic variables based on adaptive outliers. (a) Genetic turnover along each variable. (b) Accuracy and R^2^-weighted importance of all variables.

Fig. S14 Associations between environmental variables used in our study were assessed by univariable GLMs. (a) P-value of each model. Orange cells indicate a significant correlation (P < 0.05). (b) Pearson correlation coefficient (r) between variables.
